# Geometry-Dependent Tensile Load Capacity and Fracture Characteristics of Steel Wire Ropes: A Finite Element Parametric Study

**DOI:** 10.3390/ma19173671

**Published:** 2026-08-28

**Authors:** Jing Xiao, Qiqi Li, Lin Hu, Shaowei Wu, Weixiong Lin, Chengbo Gu

**Affiliations:** 1 School of Transportation, Changsha University of Science & Technology, Changsha 410114, China; 2 College of Mechanical and Vehicle Engineering, Changsha University of Science & Technology, Changsha 410114, China; 3 Dongfeng Liuzhou MOTOR Co., Ltd., Liuzhou 545026, China; 4 AISN Innovative Design and Manufacturing Co., Ltd., Liuzhou 545000, China

**Keywords:** steel wire rope, finite element analysis, wire-rope architecture, tensile energy absorption, damage modeling

## Abstract

**Highlights:**

- Baseline FEA framework was experimentally validated via 1 × 7 rope tensile tests.
- Numerical studies show geometric parameters govern load capacity and fracture modes.
- Simulations predict a 1.00 mm core diameter increases peak tensile force by 9.6%.
- Numerical models indicate that the hybrid SWR-S3 configuration achieves 99.76% TFE.
- Parameter-driven structural tailoring effectively buffers localized stress peaks.

**Abstract:**

Steel wire ropes (SWRs) are exceptional load-bearing elements. However, conventional designs often treat them as passive structures, lacking strategies to actively program their ultimate load-bearing capacity and failure behaviors. To address this gap, this study systematically investigates the tunable load capacity and fracture characteristics of SWRs by developing a simplified power-law hardening elastoplastic constitutive model and a finite element framework integrated with a ductile-damage criterion. Following material parameter calibration via single-wire tests and independent experimental validation of the baseline model using 1 × 7 strand tensile tests, comprehensive numerical parametric studies were conducted to evaluate the simulation-based influence of core diameter (*d*_core_), overall rope diameter (*D*), layer count (*F*), and strand configuration (*S*) on mechanical responses. The numerical results reveal that these geometric parameters act as effective tuning knobs that govern internal stress transfer pathways and ultimate load-bearing capacity. Specifically, simulations predict that increasing the $d_{\mathrm{core}}$ to 1.00 mm elevates the peak tensile force by 9.6% while maintaining a 90.07% tensile force efficiency (TFE, defined as the ratio of mean to peak tensile force). Furthermore, implementing a hybrid multi-strand architecture (SWR-S3) achieves an optimized TFE of 99.76%. These structural modifications facilitate internal strain synchronization, which effectively buffers localized stress peaks and dictates the progressive fracture sequence. Ultimately, this study demonstrates the potential of complementing traditional material enhancement strategies with active geometric parametrization. Rather than offering immediate industrial design rules, it provides a conceptual theoretical framework for exploring custom-tailored tensile strength profiles and predictable failure behaviors. However, because these advanced structural configurations are evaluated using idealized quasi-static finite element models, further experimental validation addressing real-world manufacturing constraints, residual stresses, and dynamic loading is required before practical engineering deployment.

## 1. Introduction

SWRs exhibit exceptional mechanical properties, including a high strength-to-weight ratio, long-distance load-bearing capacity, remarkable impact toughness, and structural stability [1,2,3,4,5]. Because of these advantages, they are widely utilized across critical applications, ranging from aerospace systems and industrial machinery to mining operations and civil engineering structures [6,7,8]. From a structural perspective, SWRs can be categorized into three main types: single-strand configurations, multi-strand designs, and composite multi-layer structures [9,10,11,12,13]. However, as operational environments become increasingly extreme, the passive load-bearing capacity of these traditional configurations is often pushed to its limits. This drives a critical need to understand and mitigate structural failure challenges, evolving conventional SWRs into highly stable architectures with tailorable energy absorption capabilities to prevent catastrophic sudden fractures.

The complex helical geometry and nonlinear contact characteristics of SWRs pose significant challenges for analytical mechanical property analysis. To address this, finite element analysis (FEA) has been widely adopted to solve complex engineering problems with promising results. Recent computational advancements have focused on capturing non-linear inter-wire friction behaviors [14,15], complex tension–torsion responses [16,17], and dynamic system-level excitations [18]. Furthermore, researchers have developed efficient modeling strategies leveraging structural periodicity [19] and chain expansion methods [20] to simulate inter-wire micro-movements and damage patterns [21,22]. While these analytical and FEA methods successfully simulate mechanical responses under given loads, they predominantly treat the SWR architecture as a fixed input rather than an adjustable design variable.

Parallel to numerical modeling, extensive experimental research has investigated the damage and fracture mechanisms of SWRs. Current experimental studies predominantly focus on observing structural degradation, such as corrosion-induced mechanical degradation [23,24,25], bending fatigue from load-specific responses [26,27,28,29], and environmental fretting wear [30,31]. For instance, structural defect analyses often link artificial wire cuts to remaining life [32] or calculate the degradation of the equivalent modulus over time [33]. While these experimental studies provide invaluable insights into physical failure mechanisms [34,35,36], they primarily treat structural fracture as an inevitable end-state to be passively observed, rather than a characteristic that can be actively controlled or delayed through initial geometric design.

Despite these extensive analytical and experimental efforts, a specific and significant unresolved design gap remains. In recent years, highly valuable frameworks have been proposed to optimize SWR structures; for instance, Filotto et al. [37] explicitly demonstrated cross-section shape optimization under tensile loading, while Liu et al. [38] developed a quantitative framework for determining optimal structural patterns in multi-strand ropes. However, there is a distinct lack of studies that simultaneously compare the effects of *d*_core_, *D*, *F*, and *S* within an experimentally calibrated ductile-damage finite element framework. Building upon these foundations, our study seeks to address this exact gap by systematically examining how these four key geometric parameters act as tuning variables to map the cross-sectional load distribution. By integrating an elastoplastic constitutive model with a ductile-damage criterion, this research offers a detailed comparative framework for transitioning SWRs to advanced architectures that mitigate severe stress concentrations and harness an ideal load plateau for enhanced structural stability.

The organization of this work is as follows: Section 2 presents the geometric structure and parametric modeling of SWRs. Section 3 details the FEA results and experimental validation. Section 4 discusses the influence of structural parameters on the tensile characteristics of SWRs, specifically investigating how cross-sectional stiffness can be tailored to mitigate stress peaks and approximate an ideal load plateau. Finally, Section 5 summarizes the conclusions of this study and highlights the paradigm shift toward mechanically tailorable rope architectures.

## 2. Establishment of Geometric Model

This section describes the parametric modeling process of SWRs, covering individual wires, strand structures, and complete rope assemblies. During the twisting process, the central steel wire in the core strand is compressed into an approximate hexagonal shape by its surroundings, which can increase the contact area, enhance fatigue strength, and improve the structural breaking load. However, to simplify the modeling in this study, we assume that its cylindrical shape remains unchanged; therefore, this localized deformation and its impact on performance are neglected. We explicitly acknowledge this as a limitation of the current idealized finite element framework. In practical engineering applications, the manufacturing process inevitably induces plastic compaction, noncircular cross-sectional distortions, residual stresses, and complex inter-wire gaps. These physical realities significantly affect localized contact stresses and often act as early initiation sites for ductile fracture. Nevertheless, because the primary objective of this study is to isolate and evaluate the macroscopic geometric scaling effects on the overall tensile load capacity, the idealized cylindrical assumption was adopted to ensure computational tractability. Future studies should integrate manufacturing-induced residual stresses and actual cross-sectional distortions to further refine these fracture predictions.

### 2.1. Parametric Modeling of the Single-Wire Spiral Line

**Figure 1:**
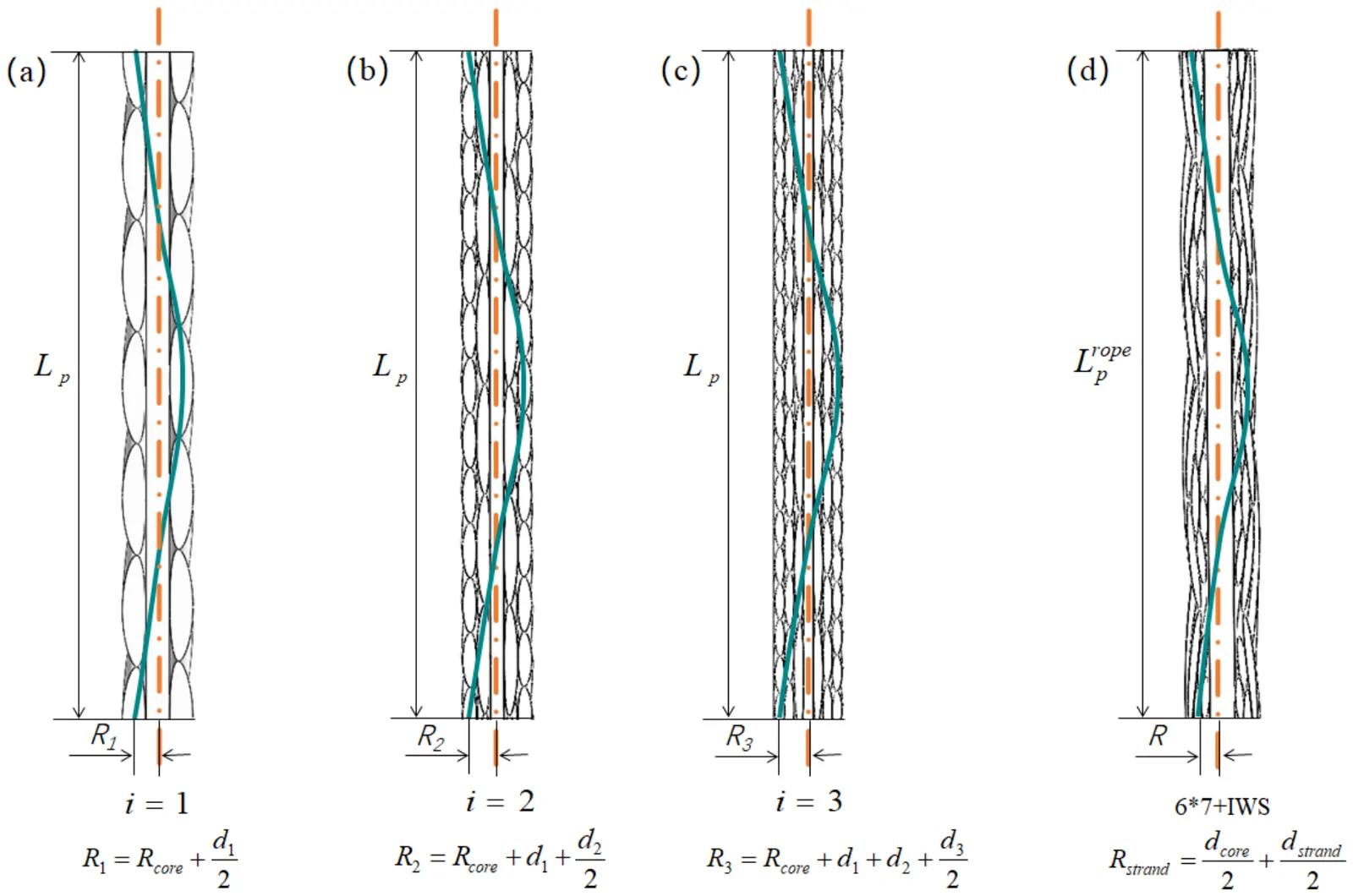
Schematic diagram of single-wire helix parameters for steel wire rope. (**a**) Helix radius of the first layer single wire. (**b**) Helix radius of the second layer single wire. (**c**) Helix radius of the third layer single wire. (**d**) Helix radius of the core wire in 6 × 7 + IWS structure.

Figure 1a–d present the helix radius of the first-layer single wire, the helix radius of the second-layer single wire, the helix radius of the third-layer single wire, and the helix radius of the core wire in the 6 × 7 + IWS structure. The axial length of one complete turn of a single wire around the core of a strand is defined as the reference twist pitch (*L*_p_). The distance from the center line of the single wire to the axis of the strand core is defined as the spiral radius (*R*). Based on these definitions, the radius of the *i*-th layer of steel wires can be derived as follows:

(1) $$R_{i}=R_{core}+\sum_{k=1}^{i-1}d_{k}+\frac{d_{i}}{2}$$

where *R*_core_ is the radius of the core strand, *d*_k_ is the diameter of the steel wires in the intermediate *k*-th layer, and *d_i_* is the diameter of the steel wires in the current *i*-th layer. It should be noted that this expression applies generally to multi-layer configurations with varying wire diameters across different layers, under the specific condition that all wires within any single given layer possess an identical diameter.

**Figure 2:**
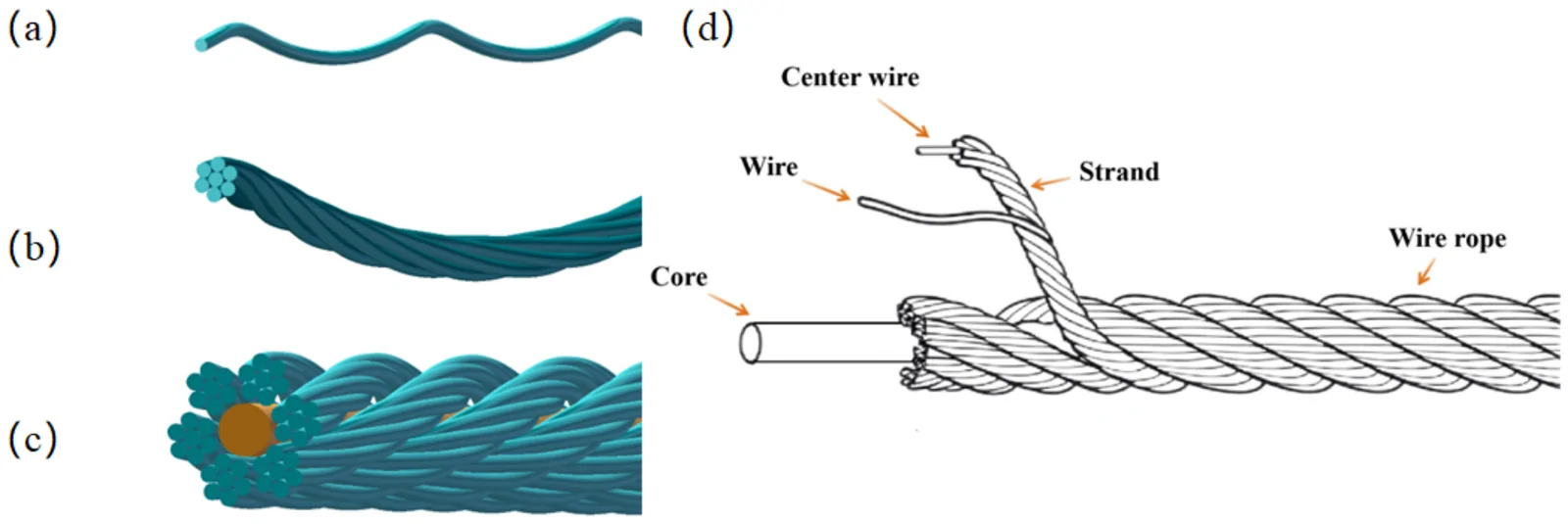
Single-wire helix of wire rope, strand structure, geometric model of 6 × 7 + IWS structure complete rope, and schematic diagram of 6 × 7 + IWS structure. (**a**) Single-wire helix model of steel wire rope. (**b**) Strand structure model of steel wire rope. (**c**) Steel wire rope model of 6 × 7 + IWS structure. (**d**) Schematic diagram of 6 × 7 + IWS structure steel wire rope.

Figure 2a shows the single-wire helix model of the SWRs. The trajectory of the center line of a single filament can be precisely described by the following parametric equations:

(2) $$r(z)=\left[\begin{array}{c} R\cos(q\frac{2\pi z}{L_{p}}+\varphi_{0})\\ R\sin(q\frac{2\pi z}{L_{p}}+\varphi_{0})\\ z \end{array}\right](0\leq z\leq L),$$

where *R* is the distance between the center line of the single wire and the axis of the strand core, *L*_p_ is the lay pitch, q is the lay twist direction of the single wire (q = 1 indicating Right lay, q = −1 indicating left lay), z is the axial coordinate,${\;\varphi }_{0}$ is the initial phase angle, and *L* is the axial length of the steel wire rope modeling section.

### 2.2. The Strand Structure of SWRs

Figure 2b shows the strand structure model of the SWRs. Taking the 6 × 7 + IWS structure as an example (six strands, seven wires per strand with an independent wire rope core), the core layer of the strand consists of one central wire (with a diameter of *d*_core_) arranged along the strand axis. The outer layer comprises six steel wires arranged in a close-packed hexagonal configuration, with a spiral radius of $R_{strand}=\frac{d_{core}}{2}+\frac{d_{strand}}{2}$.

### 2.3. Assembly of Steel Wire Rope

Figure 2c shows the steel wire rope model of the 6 × 7 + IWS structure, and Figure 2d the schematic diagram of the 6 × 7 + IWS structure steel wire rope. Six strands are spirally wound around the rope core. The trajectory equation of the center line for each strand is derived as follows:

(3) $$R(Z)=\left[\begin{array}{c} R_{strand}\cos(Q\frac{2\pi Z}{L_{p}^{rope}}+\Phi_{0})\\ R_{strand}\sin(Q\frac{2\pi Z}{L_{p}^{rope}}+\Phi_{0})\\ Z \end{array}\right],$$

where $R_{strand}$ is the distance between the center line of the strand and the axis of the rope core, $L_{p}^{rope}$ is the lay pitch of the strand structure, *Q* is the lay direction of the strand structure (*Q* = 1 indicating Right lay, *Q* = −1 indicating left lay), *Z* is the axial coordinate, and $\Phi_{0}$ is the initial phase angle of the strand structure.

To ensure the full reproducibility of the finite element models presented in this study, the exact topological and geometric parameters must be specified. For the baseline 1 × 7 and multi-strand configurations, the lay pitch of the strands was set to 6.5 times the rope diameter and the lay directions were strictly configured as detailed in Table 3, Table 5, Table 7 and Table 9. The initial phase angles $\Phi_{0}$ for the six outer strands in the baseline model were uniformly distributed at increments of 60°. Furthermore, to establish realistic inter-wire mechanics and prevent initial over-penetration errors during the dynamic explicit analysis, a microscopic initial contact clearance of 0.005 mm was applied between adjacent wires, while interference was strictly avoided in the undeformed geometric state. The exact wire diameters for all comparative models are systematically reported in the parametric analysis tables (Section 4).

## 3. Simulation Analysis and Tensile Experiments

This study comprises two independent tensile experiments, both performed using a CMT-5105GL universal testing machine (Hunan Xiangyu Measurement and Control Technology Co., Ltd., Changsha, China). The first experiment focuses on testing single steel wire to calibrate the material constitutive model, and the second one serves as an independent validation case to verify the reliability of the 1×7 steel wire rope model.

### 3.1. The Mechanical Properties of Steel Wire Rope

**Figure 3:**
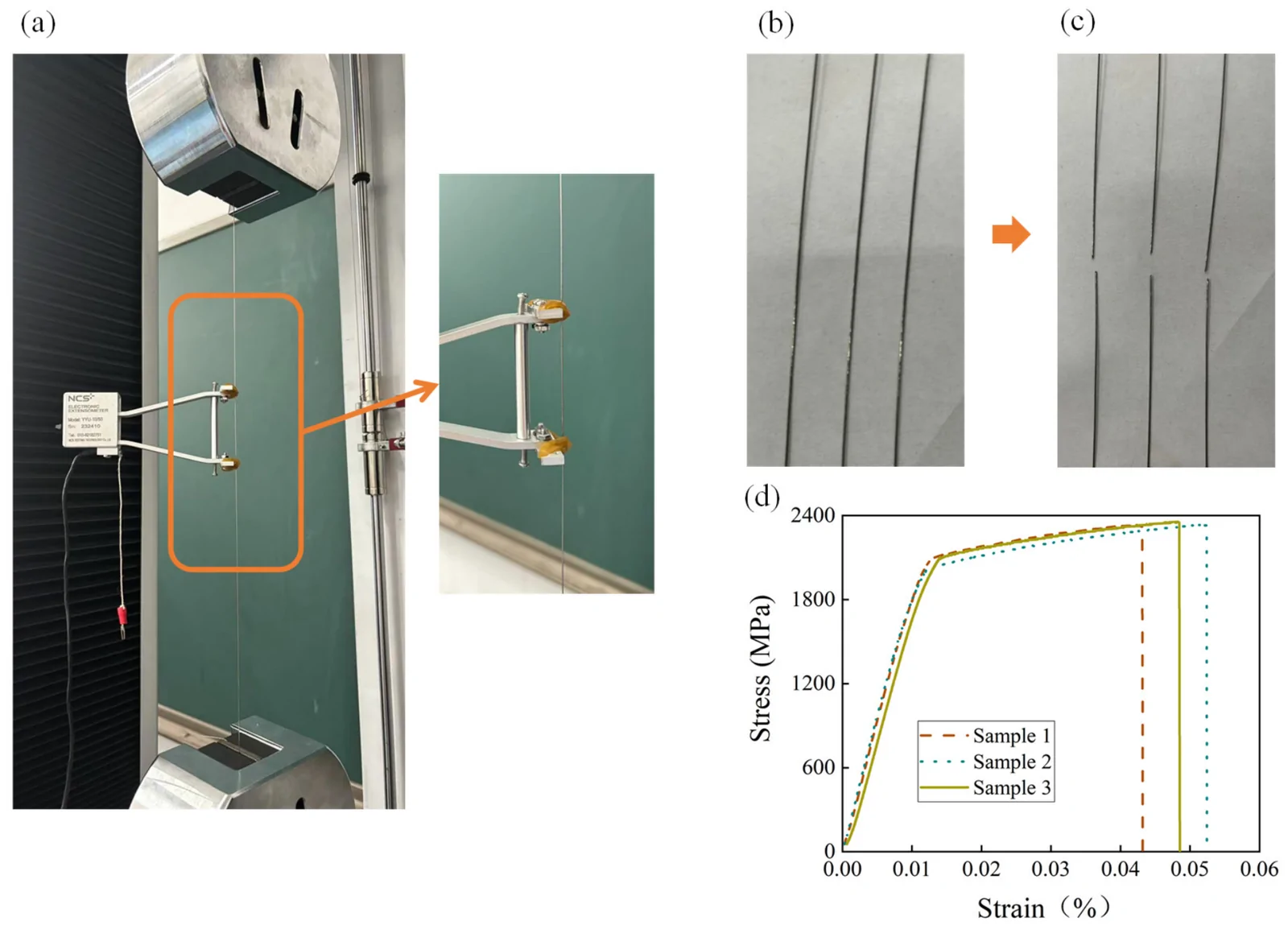
The tensile samples and tensile tests. (**a**) The universal testing machine CMT-5105GL and installation of tensile sample. The upper end of the sample is completely fixed and constrained, while the lower end retains only the degree of freedom for vertical translational motion, applying a constant speed to stretch the SWR. (**b**) Tensile samples before the experiment. (**c**) Tensile samples after the experiment. (**d**) The stress–strain curves obtained by tensile tests.

This section presents the material parameters of SWRs obtained through tensile tests on single wires. To characterize the mechanical properties of the single wires, three identical tensile samples were prepared with a length of 300 mm and a diameter of 0.48 mm. The testing apparatus and single-wire samples are shown in Figure 3a,b, with Figure 3c presenting the samples after tensile testing. Tensile tests were performed using a CMT-5105GL universal testing machine at a loading rate of 2 mm/min under room temperature conditions, and strain measurements were obtained using an extensometer. The resulting stress–strain curves are displayed in Figure 3d.

Beyond the stress–strain curve, additional material parameters were obtained. The Young’s modulus (*E*) of the steel wire was determined directly by extracting the slope of the initial linear elastic region from the experimental stress–strain curves using linear regression. According to Hooke’s law in the small-strain elastic regime, this elastic-slope method is mathematically formulated as

(4) $$E=\frac{d\sigma }{d\varepsilon }\approx \frac{\Delta \sigma }{\Delta \varepsilon }=\frac{\Delta F/A_{0}}{\Delta l/l_{0}},$$

where Δσ and Δε represent the stress increment and strain increment within the initial proportional limit, ΔF is the corresponding tensile force increment, Δl is the gauge section elongation recorded by the extensometer, $l_{0}$ is the initial gauge length ($l_{0}=50\;\mathrm{mm}$), and $A_{0}$ is the initial circular cross-sectional area of the single steel wire ($A_{0}=\pi d^{2}/4$, with wire diameter d=0.48 mm).

The density of the wire rope is 7.85 × 10^3^ kg/m^3^, and according to Equation (4), the Young’s modulus of the steel wire is calculated as 174.968 GPa. For this elastic regime, a standard Poisson’s ratio of 0.30 was assigned for the steel material. The incompressibility assumption (constant volume) was strictly reserved for formulating the subsequent plastic deformation and yielding behavior, ensuring the physical accuracy of the material model across different mechanical regimes.

In addition to the power-law hardening constitutive model, the material parameter definition in ABAQUS requires specification of both an initial damage criterion and a damage evolution law to capture material separation. To accurately simulate the damage transition and capture the distinct tensile fracture characteristics of SWRs under tensile overload, this study purposely employs the ductile-damage criterion [39]. The implementation of this criterion is essential for tracing the precise moment localized yielding translates into a predictable, tunable macroscopic fracture. The ductile criterion represents a phenomenological model that predicts damage initiation resulting from void nucleation, growth, and coalescence within the material matrix. The model formulates the equivalent plastic strain at damage initiation (${\bar{\varepsilon }}_{D}^{pl}$) as a function of stress triaxiality (η) and strain rate (${\dot{\bar{\varepsilon }}}^{pl}$), as expressed in Equation (5). Damage initiation occurs when the condition in Equation (6) is satisfied, with subsequent evolution calculated incrementally according to Equation (7).

(5) $${\bar{\varepsilon }}_{D}^{pl}\left(\eta ,{\bar{\dot{\varepsilon }}}^{pl}\right),$$

(6) $$\omega_{D}=\int \frac{d{\bar{\varepsilon }}^{pl}}{{\bar{\varepsilon }}_{D}^{pl}(\eta ,{\bar{\dot{\varepsilon }}}^{pl})}=1,$$

(7) $$\omega_{D}=\frac{\Delta {\bar{\varepsilon }}^{pl}}{{\bar{\varepsilon }}_{D}^{pl}(\eta ,{\bar{\dot{\varepsilon }}}^{pl})}\geq 0,$$

where η=−p/q is the stress triaxiality, p is the pressure, q is the von-Mises equivalent stress, ${\bar{\dot{\varepsilon }}}^{pl}$ is the equivalent plastic strain rate, and $\omega_{D}$ is a state variable that increases monotonically with plastic deformation.

To accurately describe the post-yield plastic behavior of the steel wire without conceptual ambiguity, an isotropic simplified power-law hardening model was employed. Because the tensile experiments were conducted under quasi-static conditions at a constant room temperature, strain-rate hardening and thermal softening effects were considered negligible. Therefore, instead of employing rate- and temperature-dependent constitutive equations, the flow stress is explicitly formulated as a function of the equivalent plastic strain:

(8) $$\sigma =A+B{\left(\varepsilon_{p}\right)}^{n},$$

where σ is the equivalent macroscopic flow stress, $\varepsilon_{p}$ is the equivalent plastic strain, A represents the initial yield stress, B is the strain-hardening coefficient, and n is the strain-hardening exponent. These plasticity parameters (A, B, and n, as summarized in Table 1) were carefully calibrated by fitting the true stress–strain data extracted from the post-yield region of the single-wire tensile experiments. By omitting rate and temperature dependencies, this formulation isolates the static strain-hardening characteristics required for our parametric analysis.

The standard ductile-damage initiation criterion for metals in ABAQUS requires specifying the equivalent plastic strain at the onset of damage ($\varepsilon_{f}$) as a function of stress triaxiality (η) and strain rate ($\dot{\varepsilon }$). These critical parameters are determined through the following constitutive equations:

(9) $$\eta =\frac{\sigma_{m}}{\sigma_{eq}}=\frac{\sigma_{1}+\sigma_{2}+\sigma_{3}}{3\sigma_{eq}},$$

(10) $$\varepsilon_{f}=ln(\frac{1}{1-R_{A}}),$$

where $\sigma_{m}$ represents the hydrostatic stress$\;\sigma_{m}=(\sigma_{1}+\sigma_{2}+\sigma_{3})/3$ and $\sigma_{eq}$ denotes the von Mises equivalent stress$\;\sigma_{eq}=\sqrt{\frac{1}{2}[{\left(\sigma_{1}-\sigma_{2}\right)}^{2}+{\left(\sigma_{2}-\sigma_{3}\right)}^{2}+{\left(\sigma_{3}-\sigma_{1}\right)}^{2}]}$, with $\sigma_{1},\;\sigma_{2}$ and $\sigma_{3}$ being the principal stresses. $R_{A}$ indicates the reduction in area. Under uniaxial tension conditions at a quasi-static strain rate ($\dot{\varepsilon }=0$), the stress triaxiality η=1/3.

Damage evolution in this context involves the removal of finite elements upon reaching predefined failure criteria, either in terms of critical displacement or fracture energy, thereby simulating material separation. For the current simulation, the critical failure displacement was specified as 0.1 mm to achieve realistic fracture propagation. This specific value was selected to correspond directly with the characteristic mesh size of 0.1 mm (as detailed in Section 3.2), which acts to regularize the fracture energy dissipation upon element deletion. Table 1 shows the material parameters of steel wire rope.

### 3.2. The Setup of FEA and Experiment

To ensure full experimental reproducibility, the precise material pedigree of the single steel wires must be specified. The specimens utilized in this study were manufactured from 304 stainless steel, conforming to the EN12385-4 [40] technical standard. The exact chemical composition (wt.%) of the material is C: 0.055, Si: 0.32, Mn: 0.95, P: 0.013, S: 0.012, Cr: 18.24, and Ni: 8.06. The surface condition of the tested wires was smooth (bright finish) without additional coating. The specific manufacturing route involved successive cold-drawing processes to achieve the required microstructural and mechanical baseline. Since specific initial residual stresses and pre-strain fields induced by the manufacturing process were not explicitly measured, they were treated as negligible baseline conditions in the initial state of the finite element models prior to the tensile tests.

**Figure 4:**
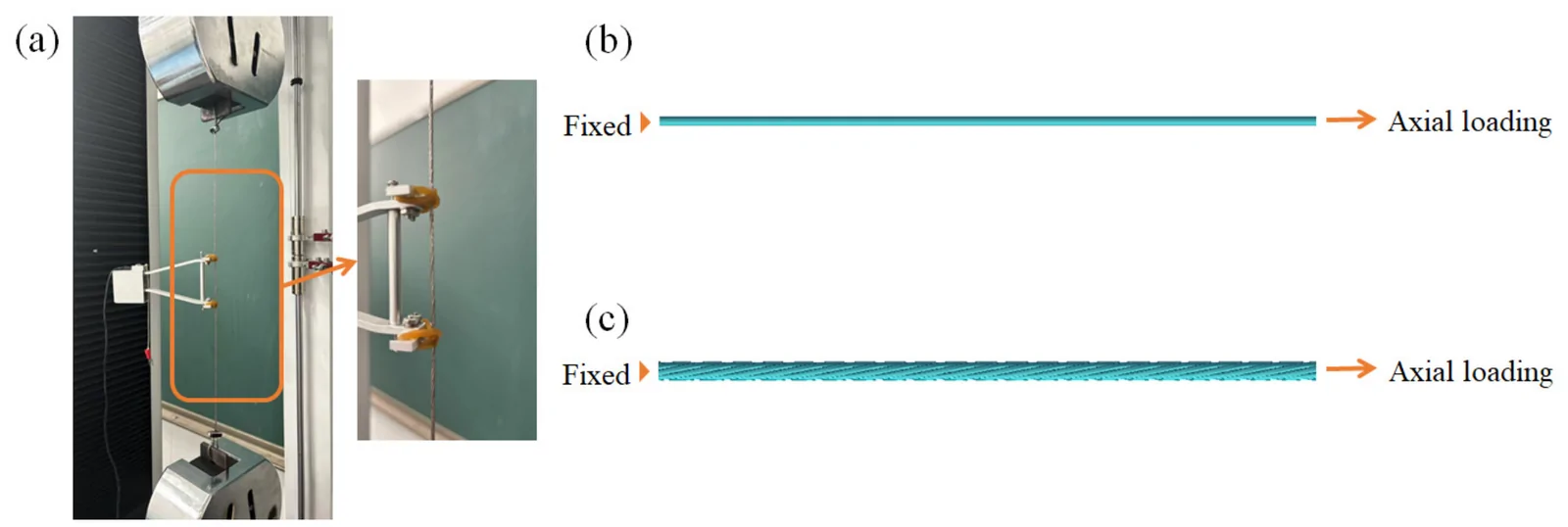
Tensile test of 1 × 7 steel wire rope sample. Finite element models of the single wire and 1 × 7 steel wire rope. (**a**) Tensile test of 1 × 7 steel wire rope. (**b**) Finite element model of the single wire. (**c**) Finite element model of 1 × 7 steel wire rope.

Figure 3a and Figure 4a present the tensile testing results of the single wire and 1 × 7 steel wire rope samples, respectively. To ensure absolute reproducibility, all testing procedures were conducted strictly in accordance with the GB/T 8358-2023 [41] standard using a CMT-5105GL universal testing machine. To prevent slippage and localized stress concentrations at the clamped ends, the specimens were secured using the direct clamping method with wedge grips. Prior to formal data acquisition, a minimal preload was applied, and the specimens were carefully aligned to eliminate bending moments and ensure pure axial loading. The specimens had a total prepared length of 300 mm, while axial deformation was precisely recorded over a 50 mm extensometer gauge length. To ensure statistical reliability and repeatability, three identical specimens were prepared and tested for both the single wire and the 1 × 7 steel wire rope configurations. All tests were conducted under strictly controlled environmental conditions at a constant room temperature of 23 ± 5 °C.

The finite element models of single steel wire and 1 × 7 steel wire rope are presented in Figure 4b,c. The models of both wires and 1 × 7 steel wire rope were constructed with identical specifications. A critical consideration for reproducibility and validation accuracy is the geometric length of the numerical model. The models were constructed with a strict axial length of 50 mm. This specific dimension was chosen to perfectly match the 50 mm gauge length of the extensometer utilized in the physical tensile tests. Aligning the numerical model length with the experimental gauge length ensures that the calculation of global strain and the accumulation of tensile energy absorption (TEA) are directly comparable, effectively eliminating scaling discrepancies or size-effect artifacts during validation. The models were meshed with C3D8R hexahedral elements using a uniform mesh size of 0.1 mm. The wire model comprised 15,984 elements, while the 1 × 7 steel wire rope model contained 108,655 elements. Numerical simulations were conducted using ABAQUS/Explicit 2020, incorporating the following computational parameters: (1) material properties including density (ρ), elastic modulus (E), Poisson’s ratio (ν), plastic stress–strain relationship, ductile-damage criterion, and damage evolution parameters. (2) A general contact algorithm to govern wire-to-wire interactions. Because inter-wire sliding resistance is central to the structural load-transfer and strain synchronization mechanisms, the tangential friction coefficient was strictly set to 0.115. This specific value is not arbitrary; it was adopted based on established experimental tribological measurements for smooth-finish, cold-drawn steel wires [14], accurately reflecting the unlubricated, bright surface condition of the experimental specimens. The normal interaction was strictly governed by a hard contact formulation to prevent unphysical inter-wire penetration. (3) Boundary conditions replicating experimental configurations, where the first end was fully constrained while the second end permitted only axial displacement. To address computational challenges inherent in explicit dynamics analysis, including extended solution durations and substantial memory demands, mass scaling was implemented with a target time increment of 1 × 10^−6^ s, maintaining the kinetic-to-internal energy ratio below 5% to ensure solution validity. Kinematic coupling constraints were applied to the end nodes to achieve uniform load distribution throughout the tensile simulation.

### 3.3. The Results of FEA and Experiments

Key energy absorption metrics analyzed in this study include tensile energy absorption (TEA), tensile specific energy absorption (TSEA), fracture energy ($G_{f}$), peak tensile force (PTF), mean tensile force (MTF), and tensile force efficiency (TFE). Their mathematical definitions are as follows:

(11) $$\mathrm{TEA}=\int_{0}^{s_{max}}F\left(s\right)\;ds,$$

(12) $$\mathrm{TSEA}=\frac{\mathrm{TEA}}{SM},$$

(13) $$G_{f}=\frac{\mathrm{TEA}}{A_{0}},$$

(14) $$\mathrm{MTF}=\frac{\mathrm{TEA}}{s_{max}},$$

(15) $$\mathrm{PTF}=\underset{0\leq s\leq s_{\max}}{\max}\left(F\left(s\right)\right),$$

(16) $$\mathrm{TFE}=\frac{\mathrm{MTF}}{\mathrm{PTF}}\times 100\%,$$

where s represents the axial displacement, $s_{max}$ is the ultimate deformation at fracture, $F\left(s\right)$ is the instantaneous tensile force as a function of displacement s, SM is the total structural mass of the model, and $A_{0}$ is the initial cross-sectional area of the steel wire rope. Based on these exact formulations, TSEA is defined as the ratio of TEA to SM, $G_{f}$ is the ratio of TEA to $A_{0}$, and MTF is the ratio of TEA to $s_{max}$. Higher TSEA and TFE values indicate that the structure possesses superior energy absorption capacity and tensile stability.

In this study, PTF and MTF serve as the primary macroscopic indicators of the SWR’s load-bearing capacity and structural breaking load. By observing the variations in these metrics across different geometric configurations, we can quantitatively assess the load-capacity tunability of the structures.

**Figure 5:**
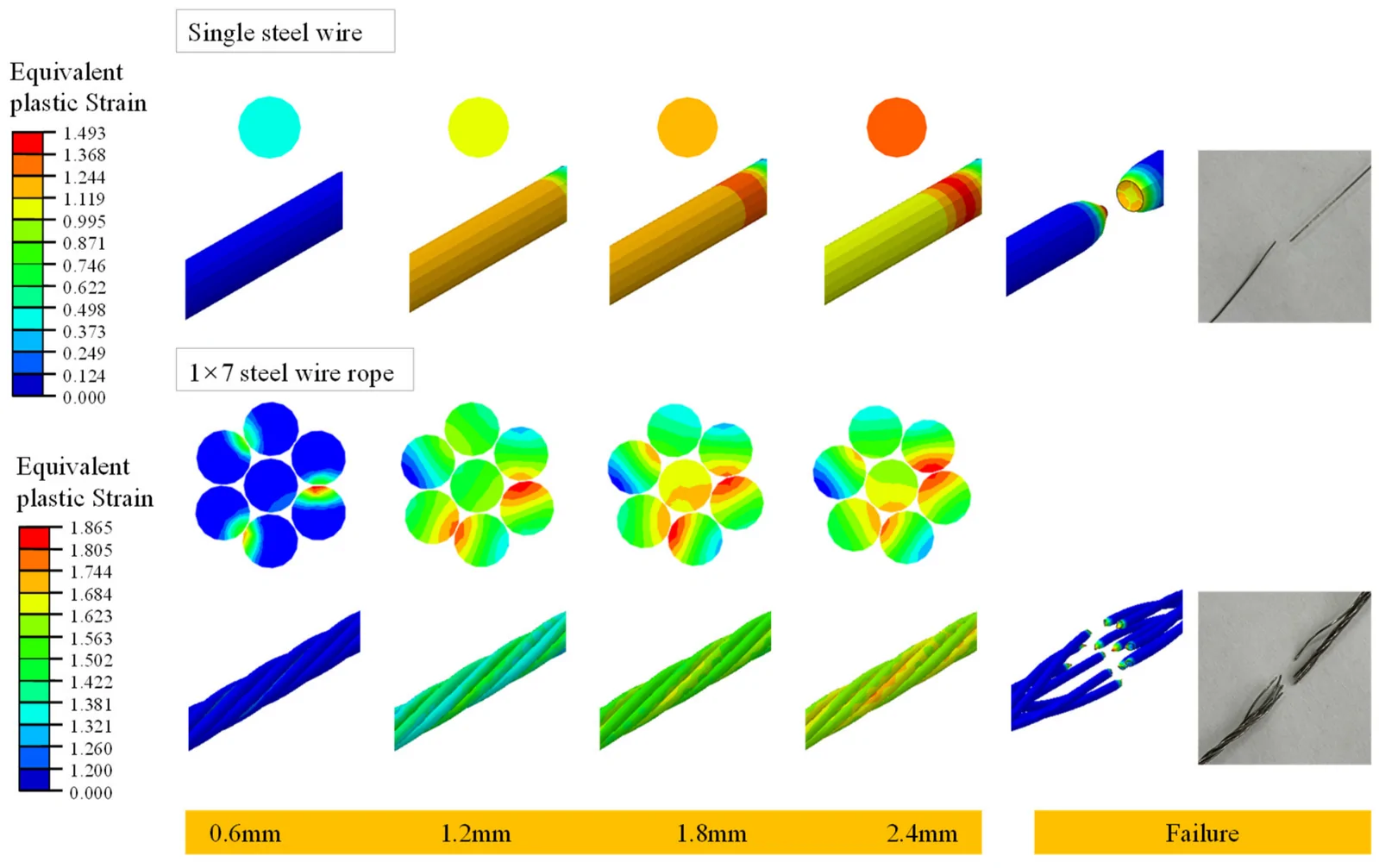
Equivalent plastic strain contour plots of single wire and 1 × 7 steel wire rope. The color scale indicates the magnitude of effective plastic strain, transitioning from blue (zero plastic strain/elastic deformation) to red (maximum plastic strain preceding ductile fracture). The plots illustrate the progressive deformation stages at displacements of 0.6 mm, 1.2 mm, 1.8 mm, 2.4 mm, and ultimate failure.

Figure 5 presents comparative equivalent plastic strain contour plots of single wire rope and 1 × 7 steel wire rope under tensile loading. The single wire rope demonstrates characteristic homogeneous deformation behavior. During initial loading (0.6 mm displacement), the strain distribution remains uniform with minimal plastic strain, consistent with elastic deformation following Hooke’s law. As displacement increases to 1.2–1.8 mm, plastic deformation initiates and accumulates as macroscopic yielding. This continuous plastic flow leads to localized strain concentrations, evident from the yellow–red color gradients, while maintaining macroscopic uniformity. At a displacement of 2.4 mm approaching fracture, the single wire rope exhibits classical macroscopic ductile failure progression: severe localized plastic yielding leads to macroscopic necking; cross-sectional reduction induces stress intensification; followed by stable plastic deformation until ultimate fracture. This sequence fully captures the predictable elastic–plastic–necking-fracture transition of homogeneous materials. In contrast, the 1 × 7 steel wire rope exhibits complex heterogeneous deformation mechanisms. Initial loading engages all wires cooperatively, but structural asymmetry creates differential strain distribution. Outer wires experience greater cumulative strain due to their helical geometry, contact stresses from pre-tension, and interfacial friction with the core wire. These combined effects lead to earlier plastic deformation in outer wires during intermediate loading with a displacement of 1.2–1.8 mm, while the core wire remains more constrained. The final fracture stage reveals distinct failure sequences: outer wires fracture first due to greater cumulative strain exposure and more severe stress conditions, followed by delayed core wire fracture resulting from its protected position and limited load redistribution capacity. This sequential fracture pattern exemplifies the discontinuous failure mechanisms inherent in multi-wire systems, arising from structural load partitioning and performance differentiation between components.

**Figure 6:**
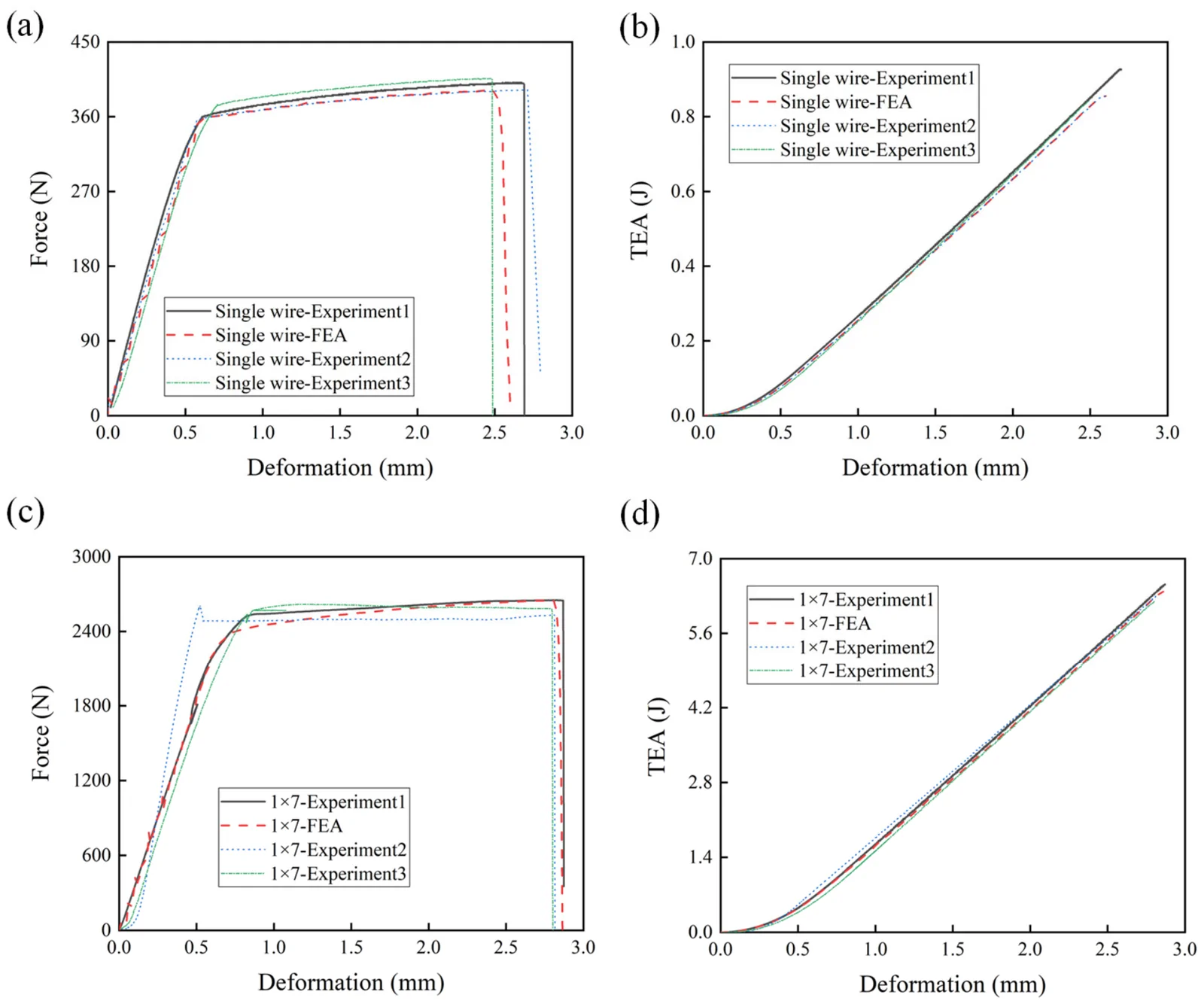
The comparison between experimental and finite element results of the single wire and 1 × 7 steel wire rope. (**a**) Force–deformation curve of single wire. (**b**) TEA–deformation curve of single wire. (**c**) Force–deformation curve of 1 × 7 steel wire rope. (**d**) TEA–deformation curve of 1 × 7 steel wire rope.

Figure 6a,c demonstrate that both the single wire and the 1 × 7 steel wire rope exhibit consistent force–deformation curves in both experimental measurements and FEA, characterized by an initial rise to peak load followed by progressive softening. This mechanical response indicates that the structures initially bear gradually increasing tensile loads until reaching maximum capacity, after which their load-bearing capability declines due to structural deformation and eventual failure. Specifically, the single wire approaches peak load at approximately 0.5–1.0 mm displacement, while the 1 × 7 steel wire rope reaches its maximum force at similar deformation levels, confirming their consistent mechanical behavior under tension. The TEA–deformation curves in Figure 6b,d reveal continuous energy dissipation throughout the tensile process for both configurations. During deformation from 0 mm to 2.5 mm, the energy absorption values increase progressively, with excellent agreement between experimental and FEA results. This close correlation validates the FEA model’s capability to accurately simulate the structures’ energy absorption mechanisms.

Table 2 presents a statistical comparison between experimental measurements and FEA. For the single wire rope, both TEA and TSEA show excellent agreement with experimental results, with a deviation of only −0.46%, while PTF demonstrates reasonable reliability with an error of −2.27%. Regarding the 1 × 7 steel wire rope configuration, TEA and TSEA maintain good consistency with an error of −2.13%, MTF exhibits a deviation of −3.83%, and PTF achieves exceptional accuracy with an error of −0.09%. This indicates high agreement in rupture force prediction. Collectively, Figure 6 and Table 2 confirm that the FEA methodology reliably simulates the mechanical performance and energy absorption characteristics of both the single wire and the 1 × 7 steel wire rope. Although certain parameters exhibit measurable deviations, the model effectively captures the essential deformation behavior, mechanical response, and energy absorption mechanisms under tensile loading.

**Figure 7:**
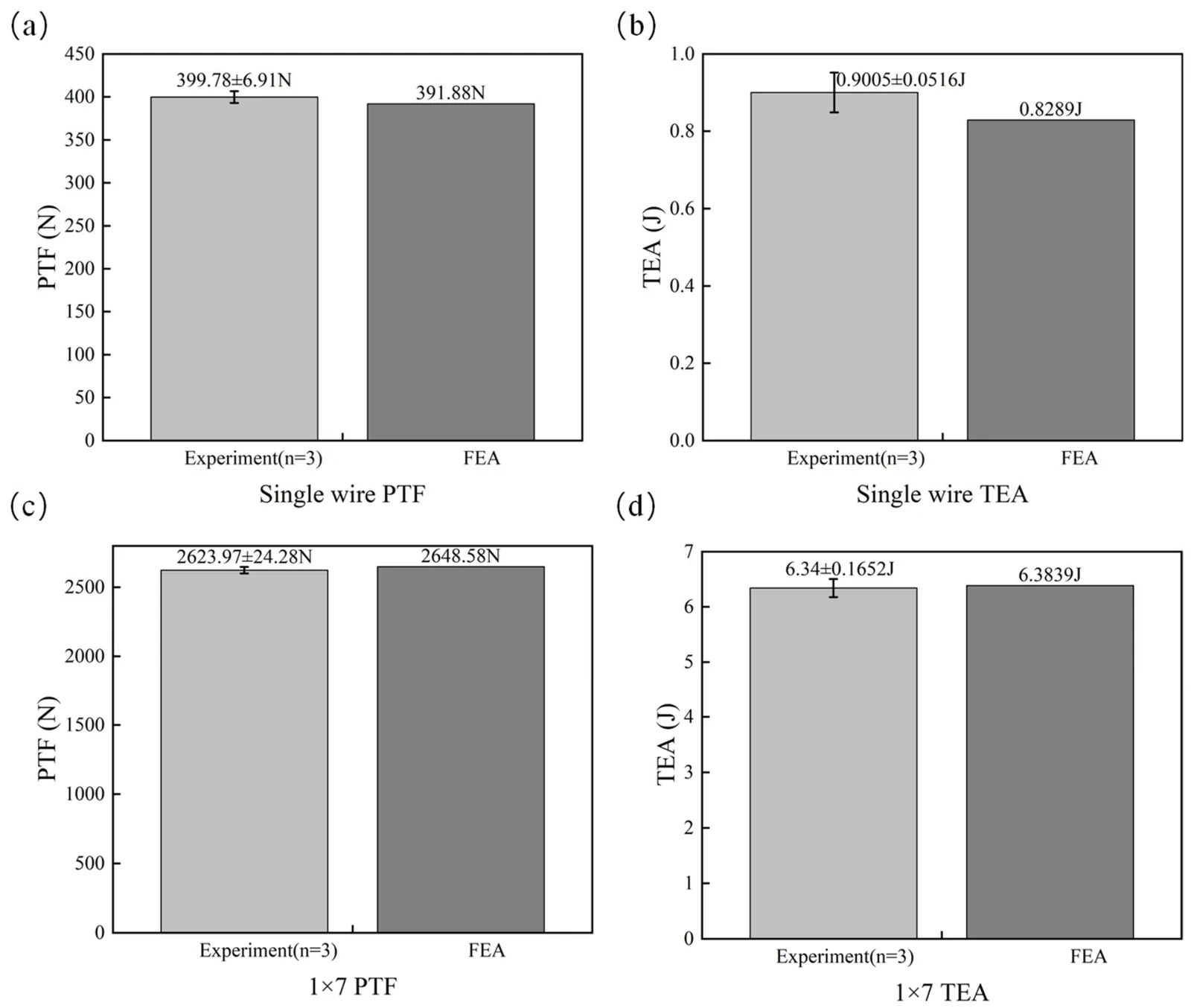
The quantitative validation of the FEA model based on repeated tensile tests: (**a**) PTF comparison of single wire, (**b**) TEA comparison of single wire, (**c**) PTF comparison of 1 × 7 steel wire rope, and (**d**) TEA comparison of 1 × 7 steel wire rope. Error bars represent standard deviations from three repeated experiments (n = 3).

To quantitatively evaluate the repeatability of the experiments and validate the accuracy of the finite element model, three repeated tensile tests were performed for both single wire and 1 × 7 steel wire rope specimens. The peak tensile force (PTF) and tensile energy absorption (TEA) were calculated from each experimental curve. The mean values and standard deviations are compared with the corresponding FEA predictions in Figure 7. The error bars represent the standard deviation obtained from repeated tests (n = 3).

## 4. Parameter Analysis of SWRs

In this section, the effects of key parameters: core diameter (*d*_core_, in mm), overall rope diameter (*D*, in mm), number of layers (*F*), and strand configuration (*S*) on the axial tensile characteristics of SWRs are explored. It must be explicitly stated that while the baseline 1×7 SWR model was physically validated in Section 3, the advanced parametric configurations presented in this section are solely numerical predictions derived from the calibrated finite element framework. Because no additional physical specimens were tested for these complex architectures, they should be interpreted as theoretical design explorations rather than experimentally verified engineering recommendations. The primary objective here is to demonstrate numerically how manipulating these variables can tune the ultimate tensile strength, facilitate a controlled fracture mechanism, and ultimately approximate an ideal load plateau under tensile loading.

### 4.1. The Mechanical Behavior of SWRs with Varying d_core_

**Figure 8:**
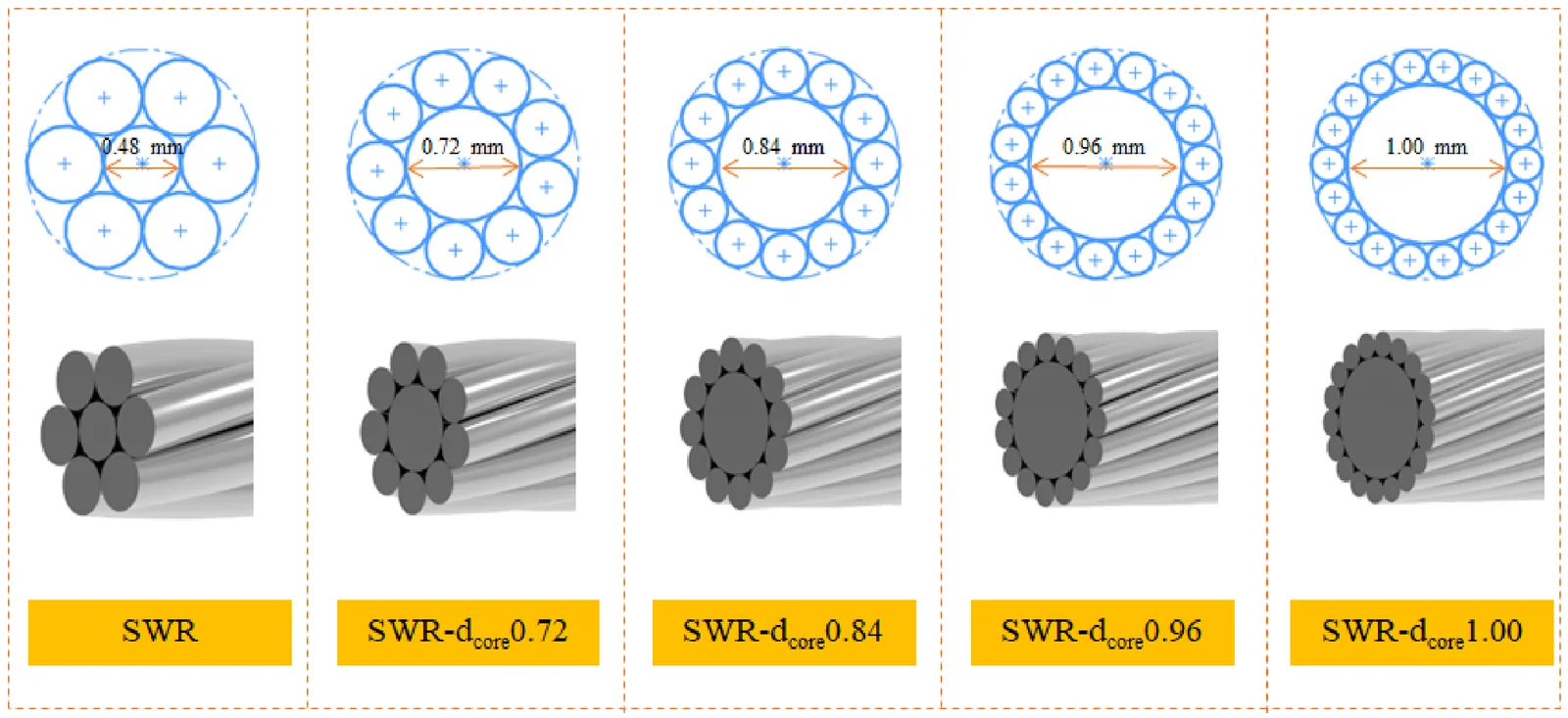
Cross-sectional views of SWR, SWR-d_core_0.72, SWR-d_core_0.84, SWR-d_core_0.96, and SWR-d_core_1.00, with *d*_core_ values of 0.48 mm, 0.72 mm, 0.84 mm, 0.96 mm, and 1.00 mm, respectively. SWR, SWR-d_core_0.72, SWR-d_core_0.84, SWR-d_core_0.96, and SWR-d_core_1.00 possess 6, 9, 12, 15, and 18 outer wires, respectively. The overall length and diameter of these steel wire rope models are 50 mm and 1.44 mm, respectively.

Five steel wire rope configurations with varying *d*_core_ were investigated while maintaining constant overall dimensions. The *d*_core_ values of these configurations are 0.48 mm, 0.72 mm, 0.84 mm, 0.96 mm and 1.00 mm. These configurations are designated as SWR, SWR-d_core_0.72, SWR-d_core_0.84, SWR-d_core_0.96, and SWR-d_core_1.00, respectively. Figure 8 displays their cross-sectional geometries, and the corresponding dimensional parameters are provided in Table 3. Rather than being mere dimensional adjustments, this parametric study reveals that varying the *d*_core_ acts as a parametric design strategy, which inherently affects two key structural characteristics. Because the total cross-sectional boundary is constrained, increasing *d*_core_ mathematically dictates two simultaneous structural alterations: an increase in the number of outer wires (*N*_outer_) and a reciprocal decrease in the diameter of these outer wires (*d*_outer_). These coupled geometric dependencies are systematically documented in Table 3 and visualized in Figure 7. Consequently, the mechanical variations observed in this specific study cannot be attributed to *d*_core_ alone; rather, they are the result of a synergistic restructuring of the entire wire architecture.

**Table 3:** The detailed dimensions of these SWR-*d*_core_ models.

	*L* (mm)	*D* (mm)	*d*_core_ (mm)	*N*_outer_ (mm)	*d*_outer_ (mm)	Twist Direction	SM (g)
SWR	50	1.44	0.48	6	0.48	Right	0.508
SWR-d_core_0.72	50	1.44	0.72	9	0.36	Right	0.530
SWR-d_core_0.84	50	1.44	0.84	12	0.29	Right	0.539
SWR-d_core_0.96	50	1.44	0.96	15	0.24	Right	0.560
SWR-d_core_1.00	50	1.44	1.00	18	0.212	Right	0.567

Note: *L* denotes the total model length, *D* represents the overall model diameter, and SM indicates the model mass.

**Figure 9:**
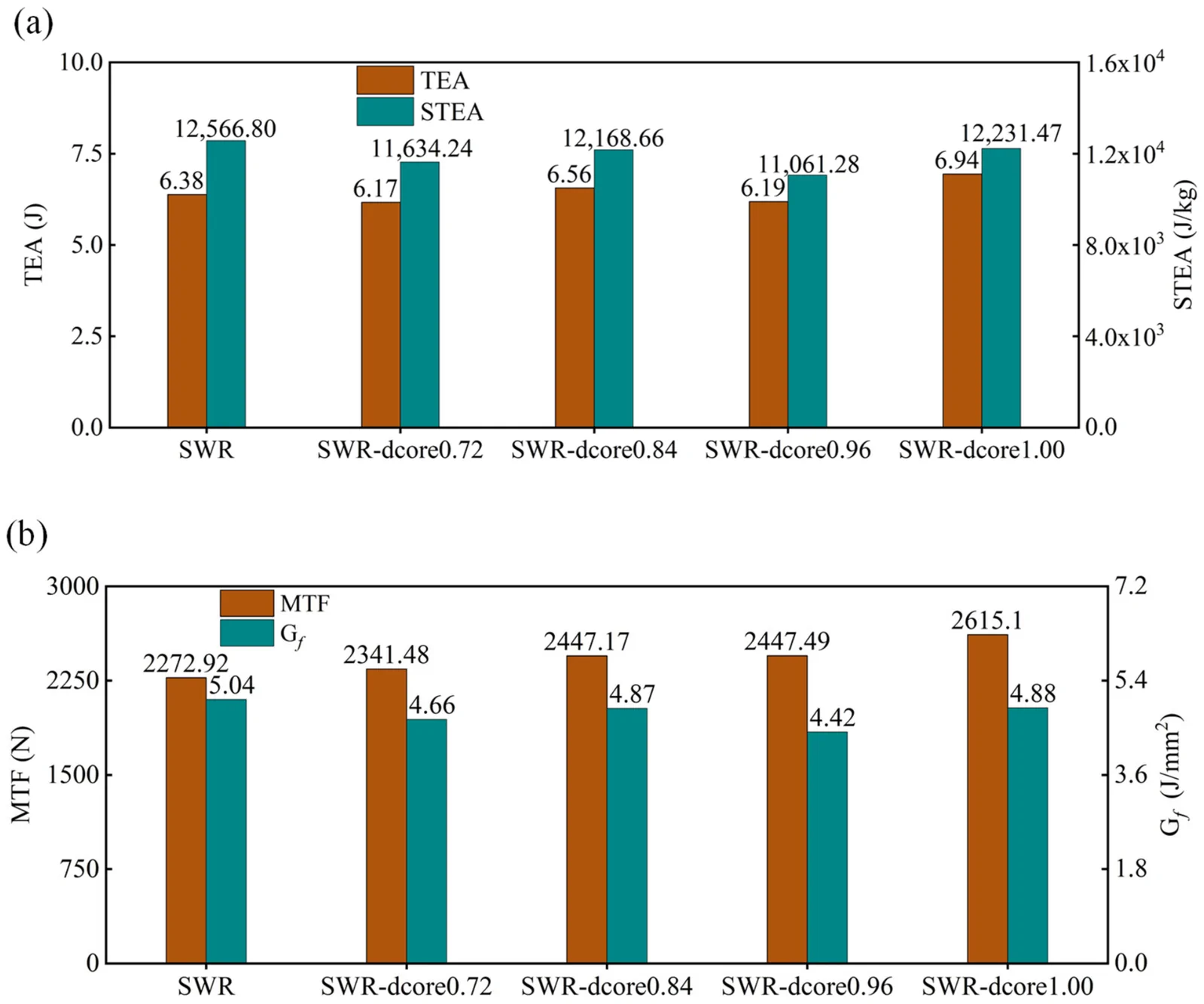
The histograms of (**a**) TEA and TSEA as well as (**b**) MTF and *G_f_* for SWR, SWR-d_core_0.72, SWR-d_core_0.84, SWR-d_core_0.96 and SWR-d_core_1.00.

Table 4 and Figure 9 present FEA results for SWR, SWR-dcore0.72, SWR-dcore0.84, SWR-dcore0.96, and SWR-dcore1.00. The FEA results demonstrate *d*_core_-dependent mechanical behavior in SWRs. When *d*_core_ increases from 0.48 mm to 1.00 mm with a D of 1.44 mm and an L of 50 mm kept constant, it produces coordinated structural changes: *N*_outer_ increases from 6 to 18, while *d*_outer_ decreases from 0.48 mm to 0.212 mm. TSEA shows a maximum efficiency of 12,566.80 J/kg for the SWR. It maintains 8.0% advantage over SWR-dcore0.72 and a 2.7% advantage over SWR-dcore1.00. This is attributable to SWR’s optimal mass-to-stiffness ratio, which is achieved by utilizing fewer outer wires with larger diameters. Both MTF and PTF increase monotonically as the configuration shifts toward larger core geometries. At a *d*_core_ of 1.00 mm, MTF reaches 2615.10 N with a 15.1% increase, and PTF reaches 2903.33 N with a 9.6% increase, respectively. Crucially, this enhanced load-bearing capacity is driven by the coupled structural effects: the enlarged core provides a significantly stiffer central backbone that resists premature yielding, while the concurrently increased number of finer outer wires (*N*_outer_ = 18) creates a denser inter-wire contact matrix. This denser matrix more effectively redistributes localized contact stresses and friction along the helical interfaces, thereby delaying macroscopic fracture and confirming the mechanical benefits of highly coupled, synergistic structural tuning. TFE remains consistently high, ranging from 85.46% to 90.07%. It peaks at 90.07% for SWR-dcore1.00, which is a 4.9% improvement over SWR. This validates optimized matching between the core and outer wires.

While increasing *d*_core_ predictably enhances absolute load-bearing capacity, evaluating normalized metrics reveals a critical trade-off in structural efficiency. As shown in Table 4, although SWR-dcore1.00 yields the highest absolute PTF (2903.33 N), its nominal ultimate stress (PTF/$A_{0}$) drops to 2043.48 MPa, which is lower than the baseline SWR’s 2090.95 MPa. Furthermore, the specific strength (PTF/SM) decreases from 5213.74 N/g in the baseline to 5120.51 N/g in the robust-core variant. Similarly, the TSEA highlights that the baseline model is more mass-efficient at absorbing energy (12,566.80 J/kg) than SWR-dcore1.00 (12,231.47 J/kg). This indicates that while a thicker core provides the necessary geometric constraint and absolute strength, it underutilizes the added cross-sectional area and mass.

**Figure 10:**
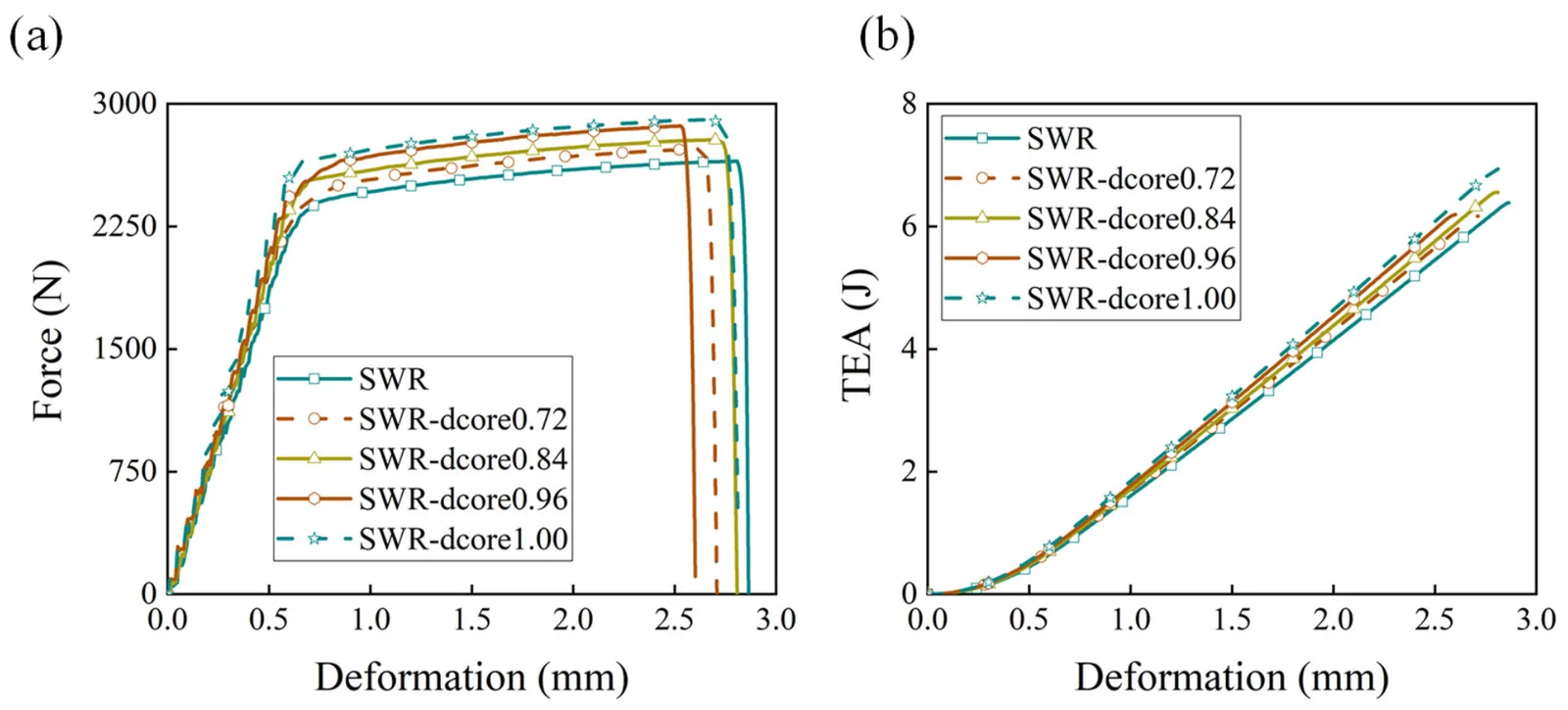
The results of (**a**) force– and (**b**) TEA–deformation curves of SWR, SWR-d_core_0.72, SWR-d_core_0.84, SWR-d_core_0.96 and SWR-d_core_1.00.

Tensile force–deformation and TEA–deformation curves for these SWR-d_core_ models are shown in Figure 10a,b. The tensile forces for SWR, SWR-d_core_0.72, SWR-d_core_0.84, SWR-d_core_0.96, and SWR-d_core_1.00 models increase approximately linearly with deformation. Notably, the baseline SWR exhibits a lower initial slope during early loading, indicating reduced initial stiffness, whereas the SWR-dcore1.00 variant shows a steeper force curve throughout, reflecting enhanced stiffness. This transition perfectly illustrates the concept of mechanically tailorable stiffness. This behavior is primarily governed by the wire rope’s tensile characteristics, which depend on d_core_ and the synergistic effect of outer wires. For wire ropes with smaller d_core_, outer wires bear most of the load; for those with larger d_core_, the inner core undertakes the major load-bearing task. In terms of energy absorption, curves for all models increase linearly, showing rapid energy absorption with deformation-evidence of good energy absorption capacity.

**Figure 11:**
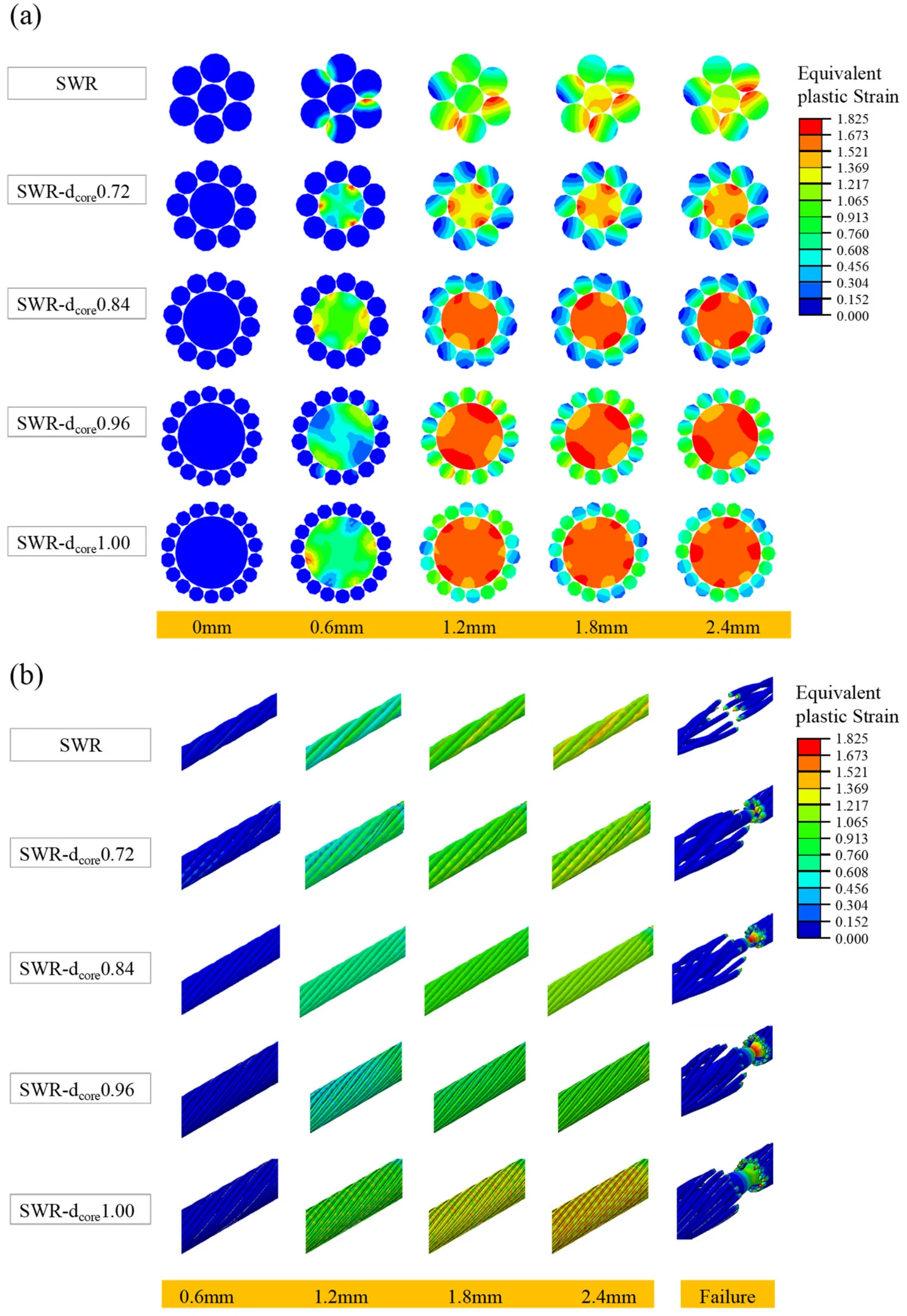
(**a**) Fracture cross-section strain distribution contours and (**b**) strain contour plots of SWR, SWR-d_core_0.72, SWR-d_core_0.84, SWR-d_core_0.96 and SWR-d_core_1.00. The color gradients represent local strain accumulation, highlighting the transition from uniform stress to localized yielding prior to fracture.

Figure 11a,b present the deformation patterns of the baseline SWR and its variants, including SWR-d_core_0.72, SWR-d_core_0.84, SWR-d_core_0.96, and SWR-d_core_1.00. They reveal how *d*_core_ governs strain evolution through structure–load–deformation coupling. The fundamental mechanism involves outer wires initiating deformation, with strain propagating inward via inter-wire contact stresses. Crucially, *d*_core_ modulates this process to tune the overall tensile strength. The smaller *d*_core_ variants SWR-dcore0.72 and SWR-dcore0.84 exhibit weak constraint, leading to an outer-dominated fracture sequence with rapid yielding, which results in lower overall strength. In contrast, the larger *d*_core_ model SWR-d_core_1.00 demonstrates enhanced geometric constraints that fundamentally alter the global strain distribution. As qualitatively observed in the equivalent plastic strain contours, the robust central core mitigates the severe, localized strain concentrations in the outer wires that are prevalent in smaller-core configurations. Rather than the outer wires absorbing the entirety of the critical deformation rapidly, the enlarged core shares a greater proportion of the structural elongation, thereby delaying the onset of critical yielding in the outer layer. This prevents premature failure and pushes the PTF upwards by 9.6%, demonstrating how *d*_core_ acts as a structural tuning parameter to redistribute internal strain pathways and elevate the structural load-bearing limits before ultimate tensile fracture.

### 4.2. The Mechanical Behavior of SWRs with Varying D

**Figure 12:**
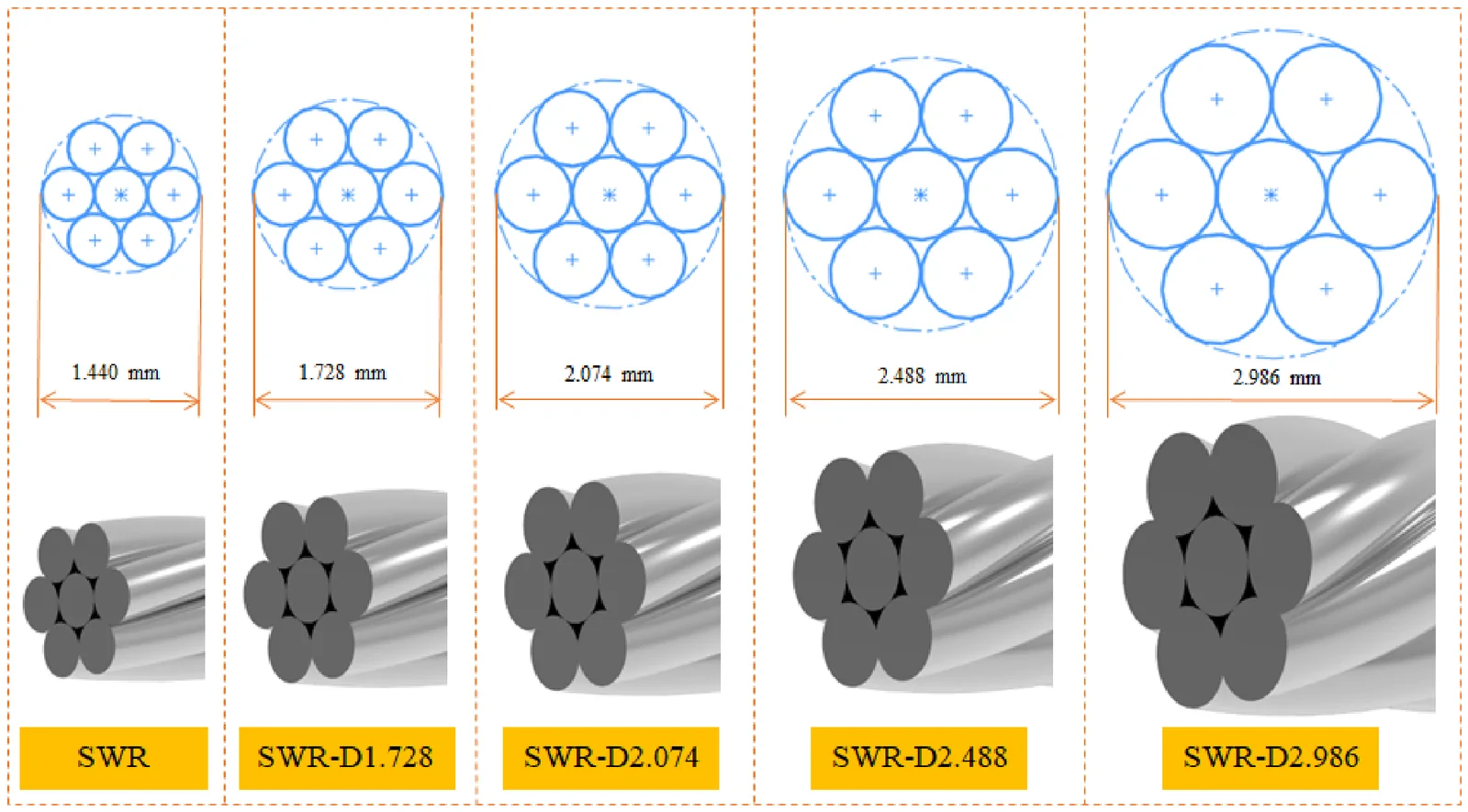
Cross-sectional views of SWR, SWR-D1.728, SWR-D2.074, SWR-D2.488, and SWR-D2.986, with D values of 1.440 mm, 1.728 mm, 2.074 mm, 2.488 mm, and 2.986 mm, respectively. The *d*_core_ values for SWR, SWR-D1.728, SWR-D2.074, SWR-D2.488, and SWR-D2.986 are 0.480 mm, 0.576mm, 0.691 mm, 0.829 mm, and 0.995 mm, respectively. The *d*_outer_ values for SWR, SWR-D1.728, SWR-D2.074, SWR-D2.488, and SWR-D2.986 are also 0.480 mm, 0.576 mm, 0.691 mm, 0.829 mm, and 0.995 mm, respectively. The overall length of these steel wire rope models is 50 mm.

This section investigates the tensile properties of SWRs with varying *D* using FEA. The evaluated *D* values are 1.440 mm, 1.728 mm, 2.074 mm, 2.488 mm, and 2.986 mm. The models are designated as SWR, SWR-D1.728, SWR-D2.074, SWR-D2.488, and SWR-D2.986. These models maintain consistent geometric proportions. This means that as *D* increases, both dcore and *d*_outer_ scale proportionally, preserving the structural configuration. The cross-sectional geometries shown in Figure 12 and dimensional details provided in Table 5 confirm this proportional scaling. This proportional scaling acts as a controlled varying parameter method, ensuring that only the absolute size varies while the relative arrangement of wires remains unchanged, thus allowing us to isolate the size-dependent effects on stiffness tailorability.

**Table 5:** The detailed dimensions of these SWR-*D* models.

	*L* (mm)	*D* (mm)	*d*_core_ (mm)	*d*_outer_ (mm)	Twist Direction	SM (g)
SWR	50	1.440	0.480	0.480	Right	0.508
SWR-D1.728	50	1.728	0.576	0.576	Right	0.737
SWR-D2.074	50	2.074	0.691	0.691	Right	1.070
SWR-D2.488	50	2.488	0.829	0.829	Right	1.570
SWR-D2.986	50	2.986	0.995	0.995	Right	2.310

**Figure 13:**
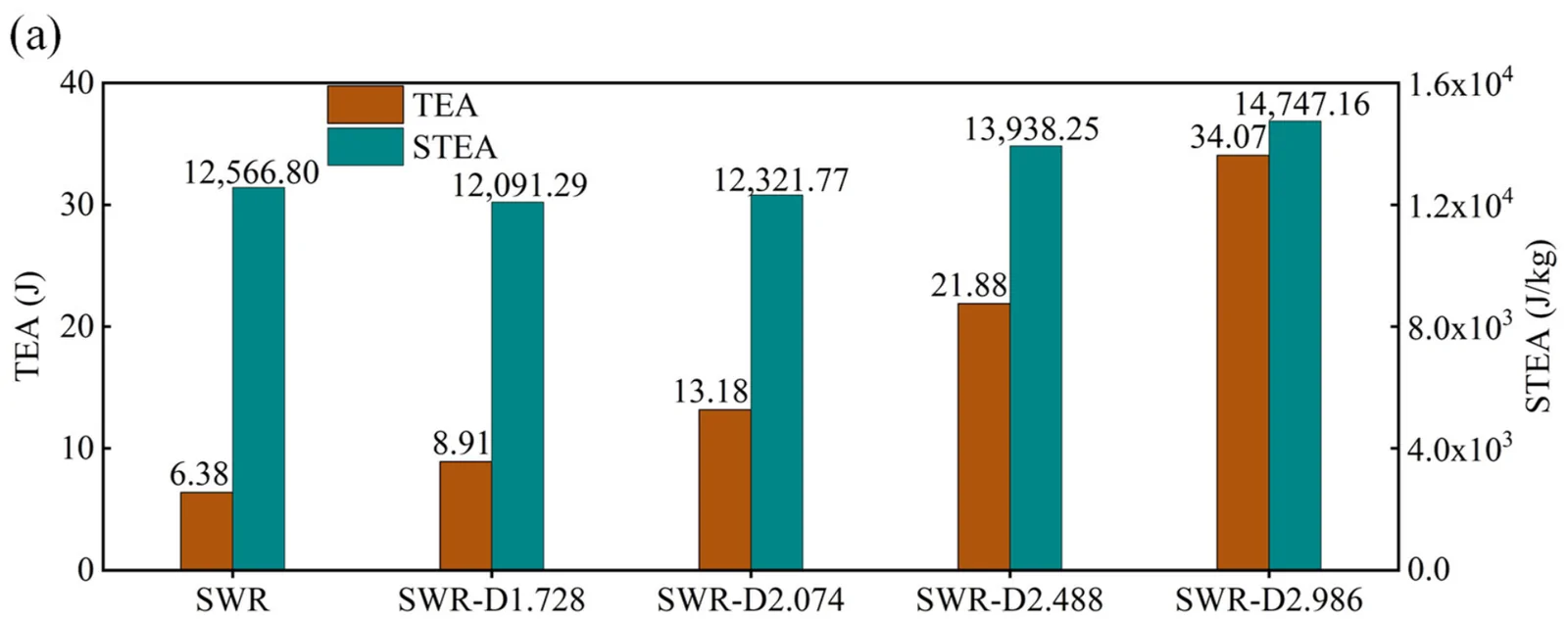

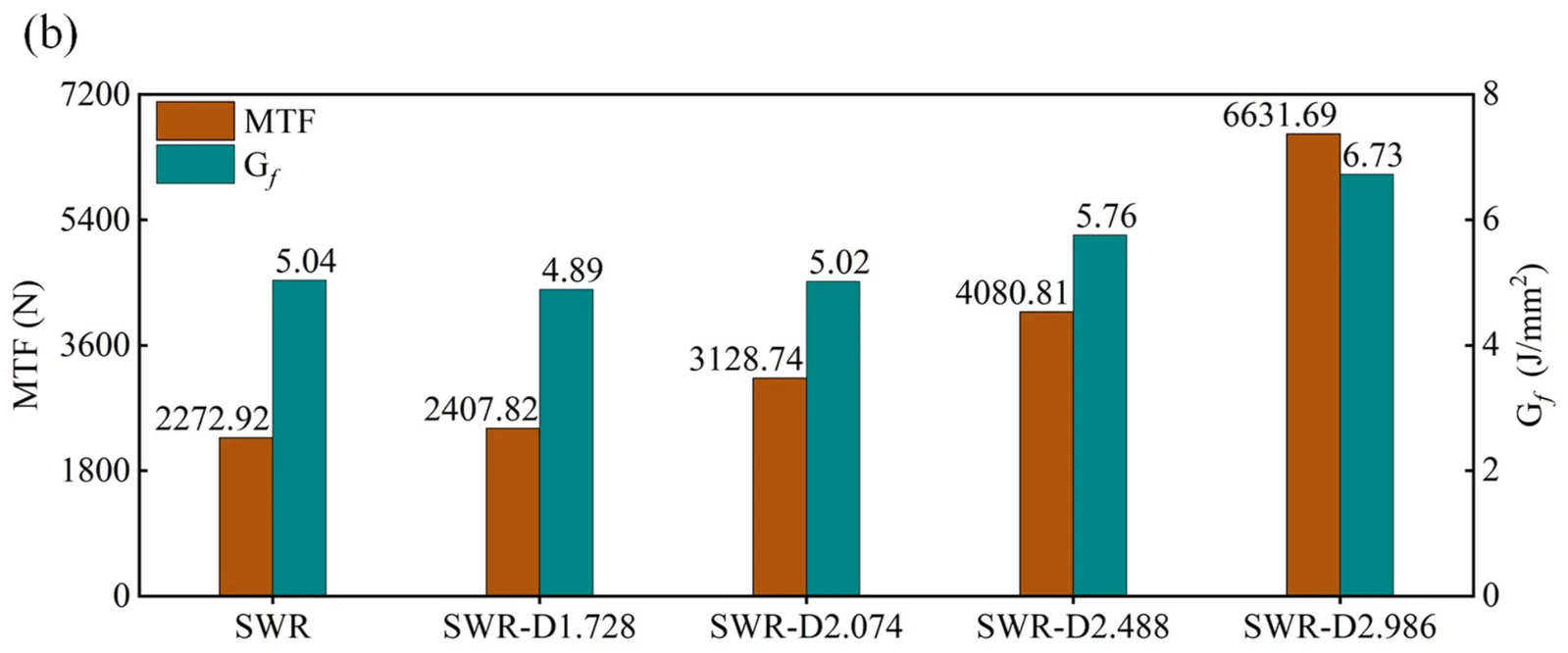
The histograms of (**a**) TEA and TSEA as well as (**b**) MTF and Gf for SWR, SWR-D1.728, SWR-D2.074, SWR-D2.488 and SWR-D2.986.

Table 6 and Figure 13 present FEA results for SWR, SWR-D1.728, SWR-D2.074, SWR-D2.488, and SWR-D2.986. The FEA systematically evaluates diameter-dependent mechanical behavior in SWRs. As *D* increases from 1.440 mm to 2.986 mm, *d*_core_ and *d*_outer_ scale coordinately. TEA shows strong diameter dependence, increasing monotonically by 434% from 6.38 J to 34.07 J. The largest *D* of 2.986 mm achieves 5.34 times the energy absorption of the SWR. TSEA exhibits nonlinear improvement, initially decreasing by 3.8% to 12,091.29 J/kg before rising to a maximum of 14,747.16 J/kg. This maximum value is 17.3% above the SWR, demonstrating superior mass efficiency despite a 355% weight increase from 0.508 g to 2.310 g. Load capacity parameters scale dramatically with diameter, showing increases of 191.7% and 284.4% in MTF and PTF, respectively. As previously noted, it should be emphasized that this substantial increase is primarily a geometric scaling effect resulting directly from the proportionally enlarged metallic cross-sectional area, rather than a change in the inherent architectural strength of the system. TFE displays distinct non-monotonic behavior, decreasing from 85.82% to a minimum of 56.51% before recovering to 65.14%. These results establish clear diameter–performance relationships: large-diameter configurations such as SWR-D2.986 optimize absolute energy absorption and load capacity. However, this absolute tensile superiority must be carefully weighed against severe practical constraints in real-world engineering applications. Increasing the overall diameter inherently incurs significant penalties regarding structural weight, reduced flexibility, and larger minimum bending radius requirements. Furthermore, thicker ropes often face increased installation complexity and a higher susceptibility to internal contact fatigue under dynamic loading. Conversely, smaller-diameter configurations such as SWR maintain advantages in force transmission stability and mass efficiency, offering superior flexibility for systems with tight spatial constraints. These findings provide definitive guidelines for application-specific design, emphasizing the necessity of balancing pure tensile strength with operational limitations.

The scaling of *D* demonstrates a profound divergence between absolute strength and specific structural efficiency (Table 6). SWR-D2.986 achieves an exceptional absolute PTF of 10,180.90 N; however, its specific strength (PTF/SM) sharply declines to 4407.32 N/g compared to the baseline’s 5213.74 N/g. The nominal stress (PTF/A_0_) similarly drops to 2012.17 MPa. Conversely, enlarging the diameter drastically improves the structure’s specific energy dissipation capabilities. The TSEA of SWR-D2.986 surges to an impressive 14,747.16 J/kg, far exceeding the baseline (12,566.80 J/kg). This demonstrates that while large-diameter configurations suffer from diminishing returns in strength-to-weight efficiency, they become highly advantageous for applications prioritizing mass-normalized energy absorption.

**Figure 14:**
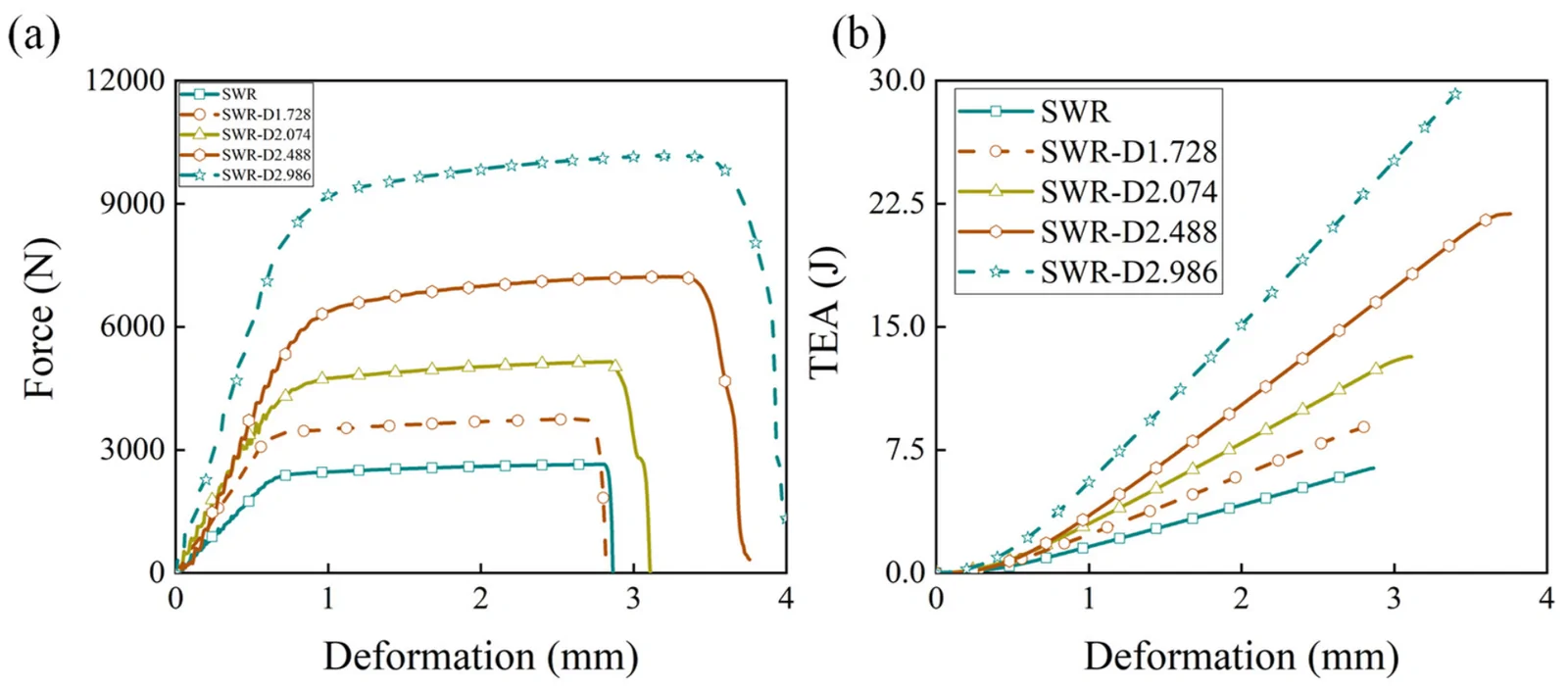
The results of (**a**) force– and (**b**) TEA–deformation curves of SWR, SWR-D1.728, SWR-D2.074, SWR-D2.488 and SWR-D2.986.

Tensile force–deformation and TEA–deformation curves for these SWR-D models are shown in Figure 14a,b. Forces for SWR, SWR-D1.728, SWR-D2.074, SWR-D2.488, and SWR-D2.986 increase approximately linearly with displacement, with SWR exhibiting a smaller initial slope that indicates lower initial stiffness. In contrast, SWR-D2.986 shows a steeper force curve during loading, demonstrating superior stiffness. This distinct transition underscores the feasibility of mechanically tailoring the structural stiffness by modulating the overall diameter. This behavior is governed by the steel wire rope’s tensile characteristics, which depend on the overall diameter *D* and synergistic load transfer between core and outer wires. Regarding energy absorption, all models’ curves increase linearly, showing rapid energy absorption with deformation-evidence of good energy absorption capacity.

To further isolate and quantify these non-trivial size effects, a theoretical scaling analysis was conducted. Under ideal proportional scaling without size effects, the peak tensile force should scale strictly with the cross-sectional area, meaning it is proportional to the square of the overall diameter ($D^{2}$). Taking the baseline SWR (*D* = 1.440 mm, PTF = 2648.58 N) as the reference, the theoretical peak tensile force ($PTF_{theo}$) for the scaled models is defined as

(17) $${PTF}_{theo}={PTF}_{baseline}\times {\left(\frac{D}{D_{baseline}}\right)}^{2}$$

Applying this baseline reveals a clear deviation in the FEA results. For instance, at the largest diameter (*D* = 2.986 mm), the ideal $D^{2}$ scaling predicts a $PTF_{theo}$ of approximately 11,386 N. However, the actual FEA prediction is 10,180.90 N, exhibiting a noticeable strength deficit of roughly 10.6% compared to the theoretical ideal. This systematic deviation from the $D^{2}$ baseline quantitatively demonstrates a structural size effect. It indicates that as the spatial scale of the wire rope increases, the complex inter-wire sliding, contact stress asymmetries, and local yielding mechanisms do not scale perfectly linearly. Instead, the enlarged helical geometry introduces slight inefficiencies in synergistic load sharing, physically validating why the structural TFE experiences a corresponding decline at larger diameters.

**Figure 15:**
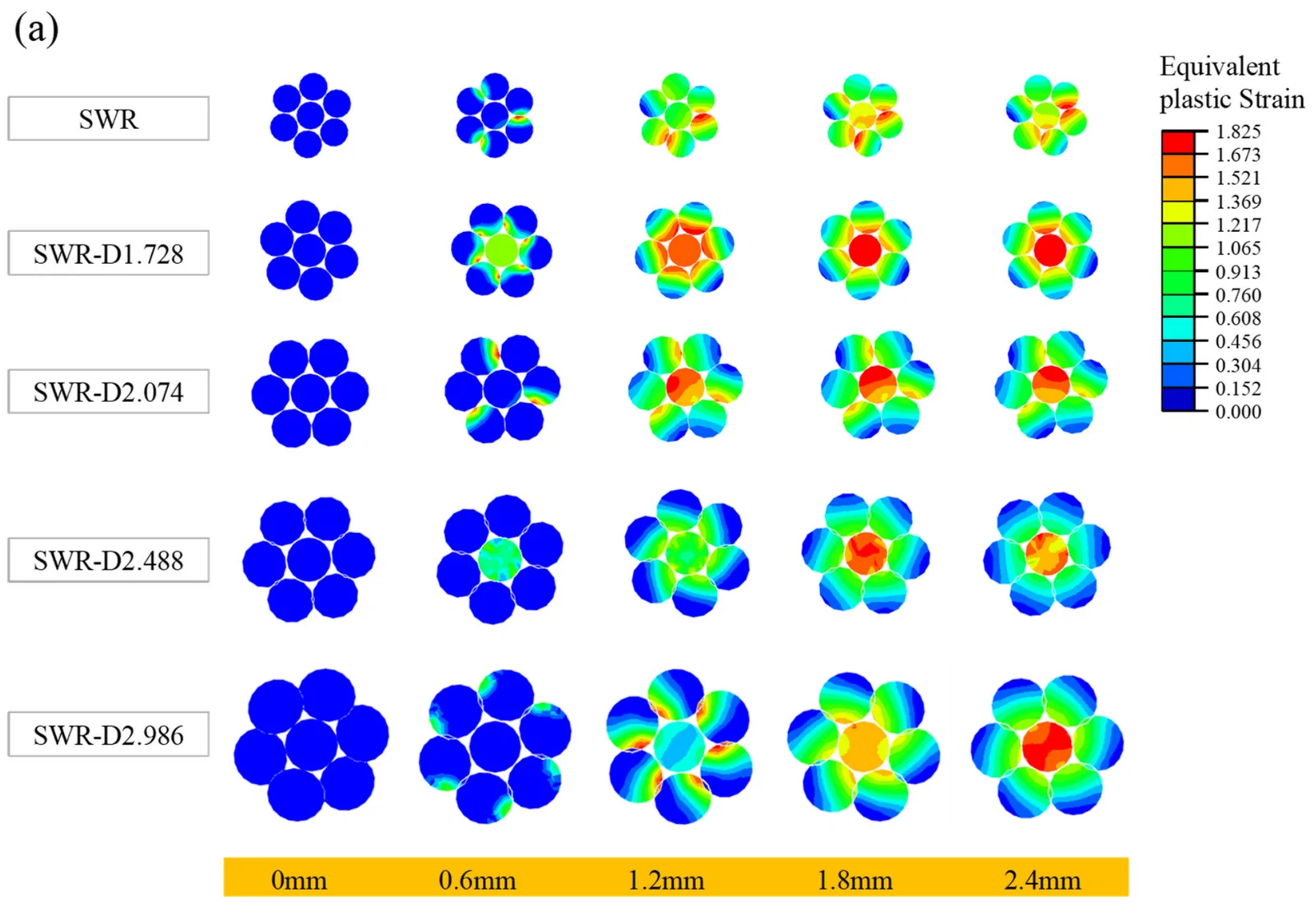

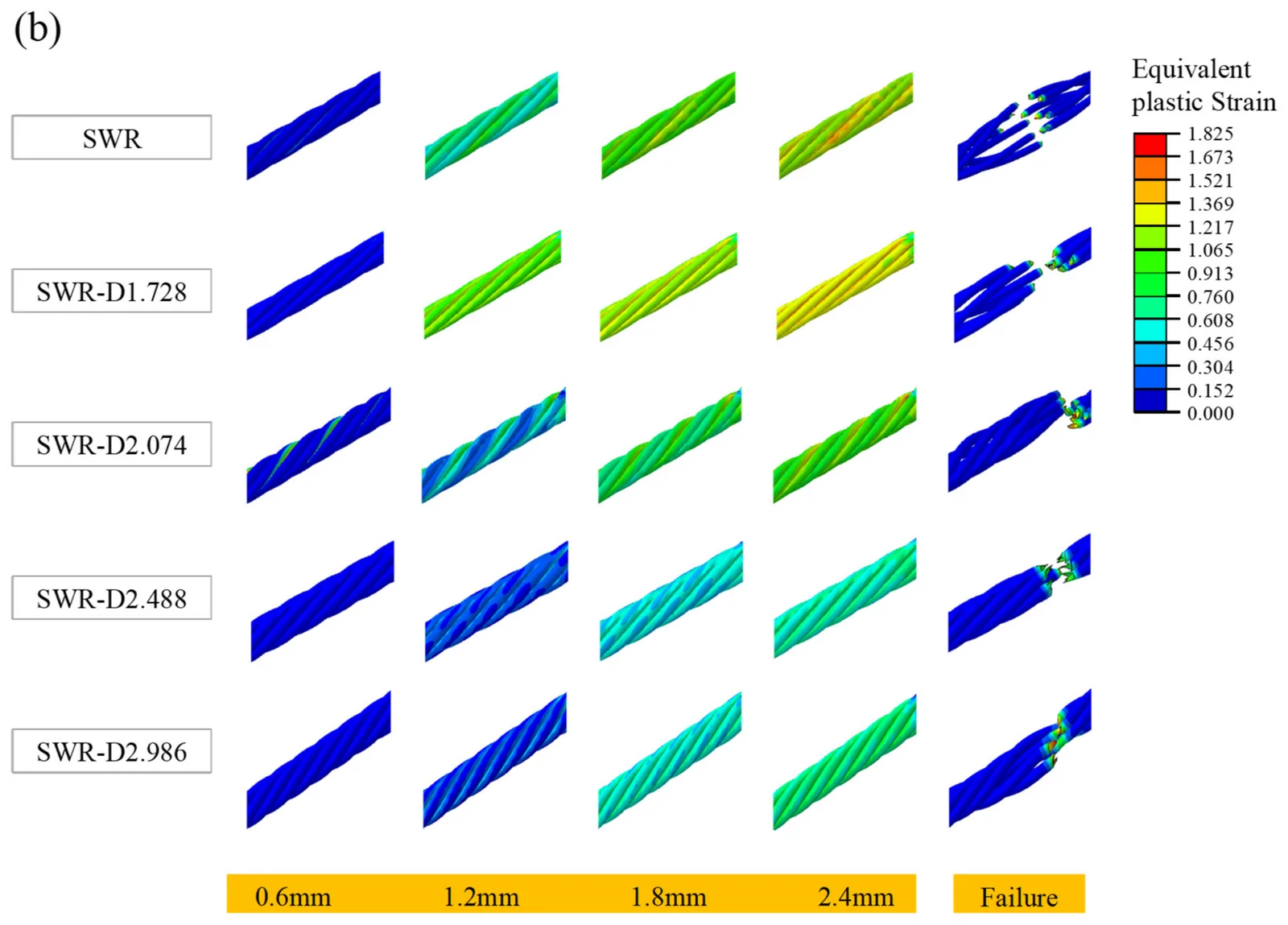
(**a**) Fracture cross-section strain distribution contours and (**b**) strain contour plots of SWR, SWR-D1.728, SWR-D2.074, SWR-D2.488 and SWR-D2.986. The color gradients represent local strain accumulation, highlighting the transition from uniform stress to localized yielding prior to fracture.

Figure 15a,b present the deformation patterns of SWR and its diameter variants including SWR-D1.728, SWR-D2.074, SWR-D2.488, and SWR-D2.986. They demonstrate how *D* governs strain evolution through coupled surface effects and structural constraints. Small-diameter ropes exhibit rapid strain synchronization due to weak constraints, enabling outer-initiated yielding with quick core penetration. In contrast, large-diameter ropes develop a rigid core matrix that initially buffers deformation. This dimensional dependence follows a clear mechanism for size-driven strength scaling and fracture tuning. *D* governs contact constraints, which in turn dictates the structural tensile load capacity. Small diameters achieve rapid inter-wire synchronization, while large diameters activate a core-driven buffering mechanism that delays catastrophic failure. This observation correlates precisely with the dramatic 284.4% increase in PTF, demonstrating that dimensional scaling is a highly effective geometric lever for tuning the absolute load-bearing capacity of SWRs to meet extreme heavy-load demands.

### 4.3. The Mechanical Behavior of SWRs with Varying F

**Figure 16:**
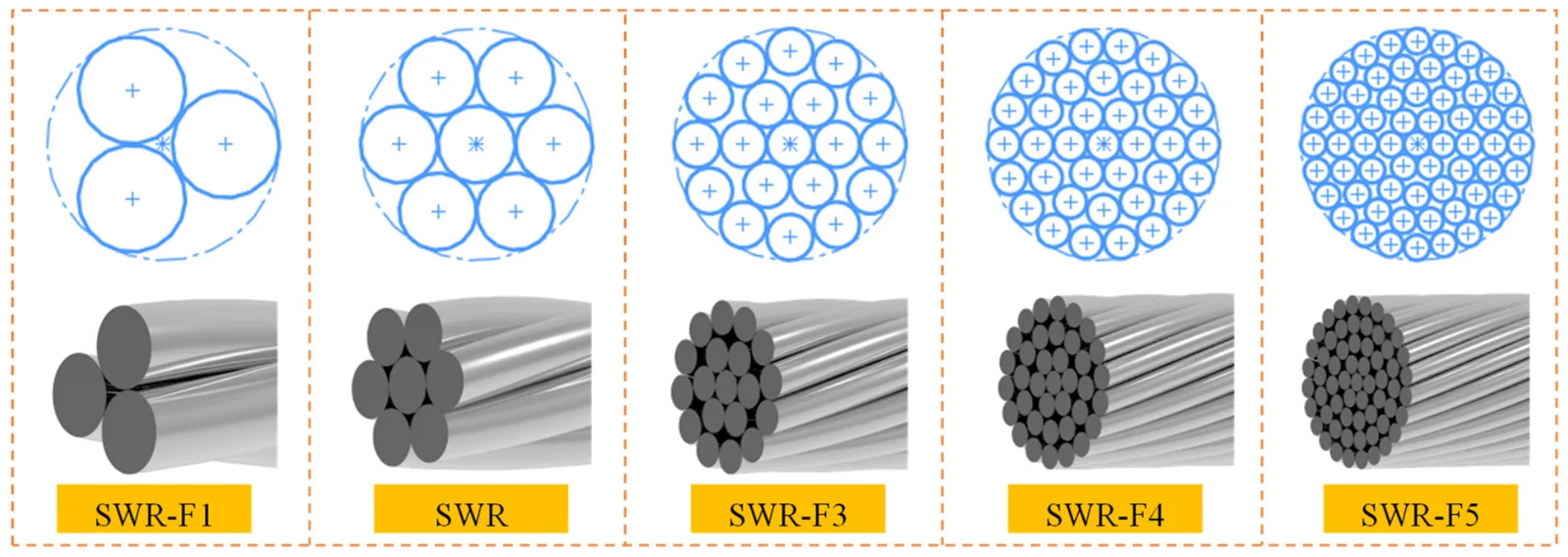
SWR-F1 is composed of 3 wires twisted in a single layer, SWR consists of 7 wires twisted in two layers with a wire count structure of 1 + 6 per layer, SWR-F3 comprises 19 wires twisted in three layers with a wire count structure of 1 + 6 + 12 per layer, SWR-F4 is made up of 37 wires twisted in four layers with a wire count structure of 1 + 6 + 12 + 18 per layer, and SWR-F5 consists of 61 wires twisted in five layers with a wire count structure of 1 + 6 + 12 + 18 + 24 per layer. Their respective F-values are 1, 2, 3, 4, and 5.

This section analyzes the tensile characteristics of SWRs with a varying *F* while maintaining constant overall dimensions. The numerical models are designated as SWR-F1, SWR, SWR-F3, SWR-F4 and SWR-F5 based on their layer count. These models exhibit systematic variations in both *d*_core_ and *d*_outer_. This is evidenced by their cross-sectional geometries shown in Figure 16 and dimensional specifications provided in Table 7. The parametric study demonstrates that increasing the layer number fundamentally modifies the rope’s structural architecture through coordinated adjustments of these key geometric parameters. This multi-layer approach serves as a hierarchical programming strategy, allowing for the active redistribution of internal load-bearing pathways to tailor the overall structural stiffness.

**Table 7:** The detailed dimensions of these SWR-*F* models.

	*L* (mm)	*D* (mm)	*F*	*d* (mm)	Twist Direction	SM (g)
SWR-F1	50	1.44	1	0.664	Right	0.414
SWR	50	1.44	2	0.48	Right	0.508
SWR-F3	50	1.44	3	0.288	Alternate lay	0.498
SWR-F4	50	1.44	4	0.2056	Alternate lay	0.494
SWR-F5	50	1.44	5	0.16	Alternate lay	0.493

**Figure 17:**
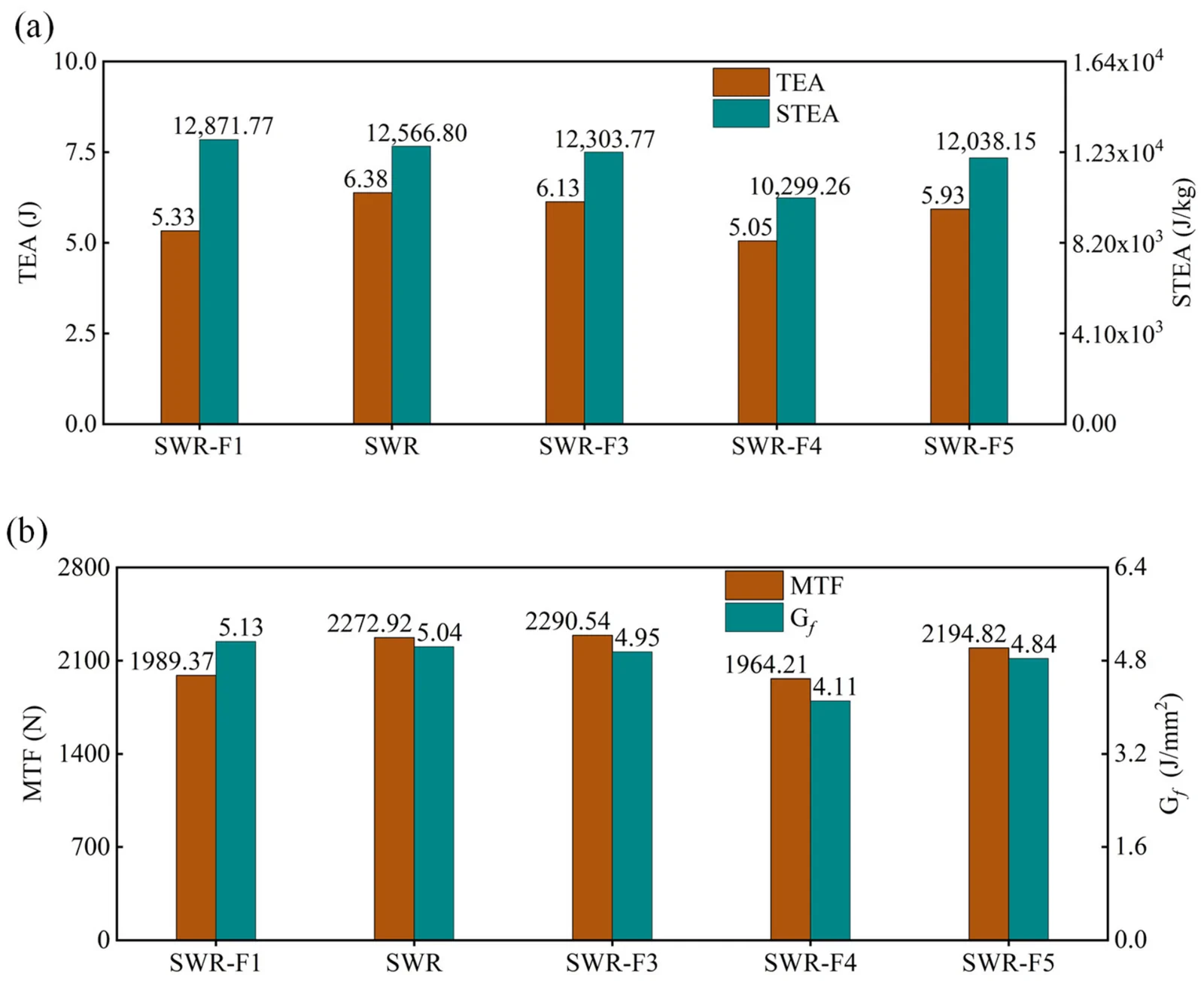
The histograms of (**a**) TEA and TSEA as well as (**b**) MTF and Gf for SWR-F1, SWR, SWR-F3, SWR-F4 and SWR-F5.

Table 8 and Figure 17 present FEA results for SWR-F1, SWR, SWR-F3, SWR-F4, and SWR-F5. FEA was conducted on five layered steel wire rope configurations. All configurations maintain a constant overall diameter *D* of 1.44 mm and a length *L* of 50 mm. As the layer count increases, the single-wire diameter *d*s decreases from 0.664 mm to 0.16 mm. The twist direction transitions from Right to Alternate lay when the layer count *F* is 3 or higher. TEA peaks at 6.38 J for the two-layer configuration SWR, which is 19.8% higher than the single-layer configuration. TSEA maximizes at 12,871.77 J/kg for the single-layer design, 6.9% higher than the five-layer configuration. MTF optimizes at 2290.54 N in the three-layer structure, and PTF optimizes at 2648.58 N in the two-layer structure. TFE reaches 93.89% in the five-layer configuration, a 9.40% improvement over the two-layer configuration. This demonstrates that two-layer structures achieve an optimal balance between energy absorption of 6.383 J and ultimate load capacity of 2648.58 N. Single-layer designs provide superior mass efficiency of 12,871.77 J/kg. Five-layer Alternate lay configurations enable the most stable force transmission of 93.89%. These findings support performance-driven layer selection for specific engineering applications.

Analyzing the normalized metrics for multi-layer configurations (Table 8) provides deeper insight into their mechanical behavior. The simpler SWR-F1 configuration demonstrates the highest specific strength (5355.02 N/g) and TSEA (12,871.77 J/kg) in this series. As layer complexity increases to SWR-F5, the specific strength drops to 4741.89 N/g and the nominal stress (PTF/$A_{0}$) falls to 1906.07 MPa. However, despite this reduction in absolute strength-to-weight ratio, SWR-F5 maintains a robust TSEA of 12,038.15 J/kg alongside an outstanding force transmission efficiency (TFE) of 93.89%. This confirms that the primary advantage of high-layer-count configurations lies not in specific strength, but in superior load distribution and stable, synchronized energy dissipation across a dense internal architecture.

**Figure 18:**
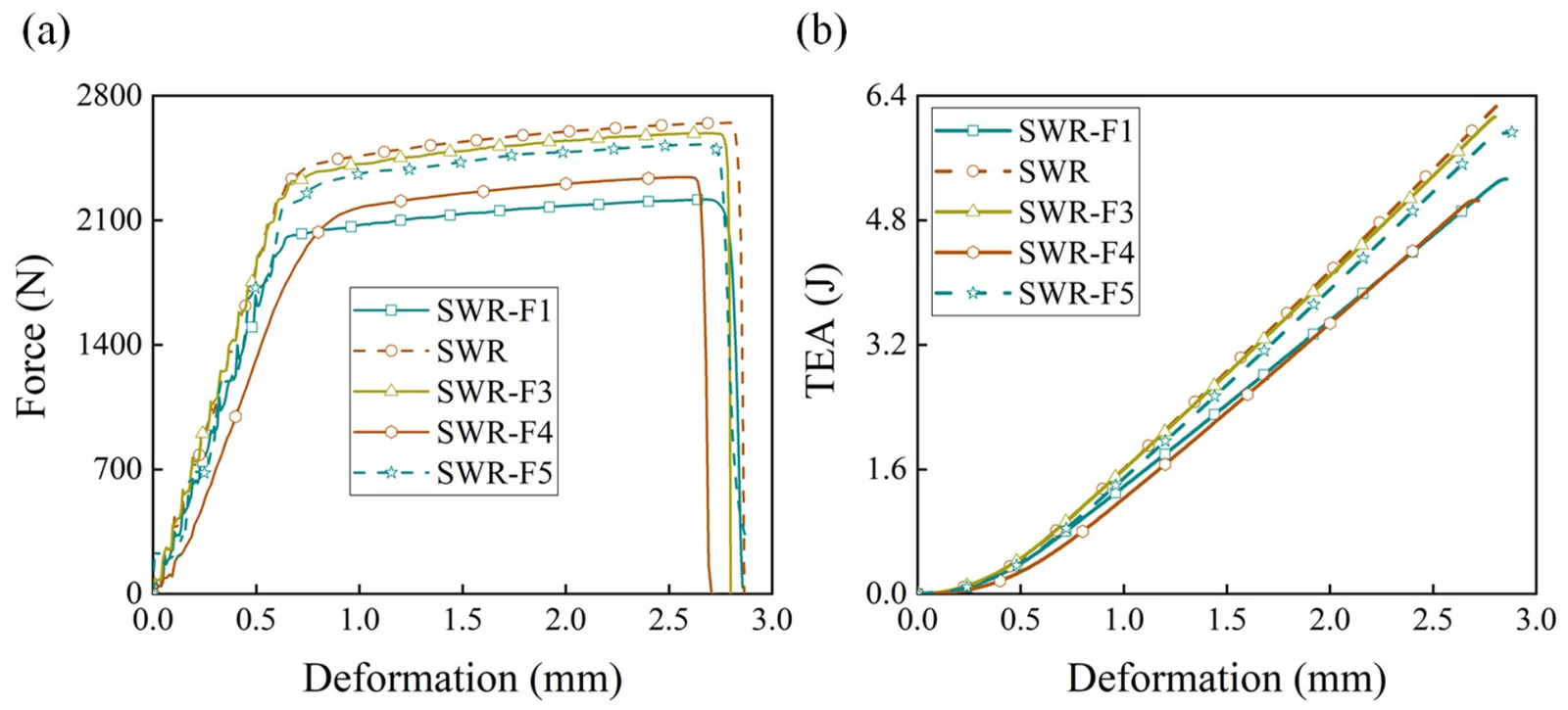
The results of (**a**) force– and (**b**) TEA–deformation curves of SWR-F1, SWR, SWR-F3, SWR-F4 and SWR-F5.

Tensile force–deformation and TEA–deformation curves for these SWR-F models are shown in Figure 18a,b. SWR-F4 exhibits a smaller initial slope and lower initial stiffness. In contrast, SWR-F3 shows a steeper force curve during loading, indicating superior stiffness. This sharp contrast underscores the capacity to mechanically tailor the rope’s yielding behavior by F. For energy absorption, all the models’ curves increase linearly, showing rapid energy absorption with deformation-evidence of good energy absorption capacity.

**Figure 19:**
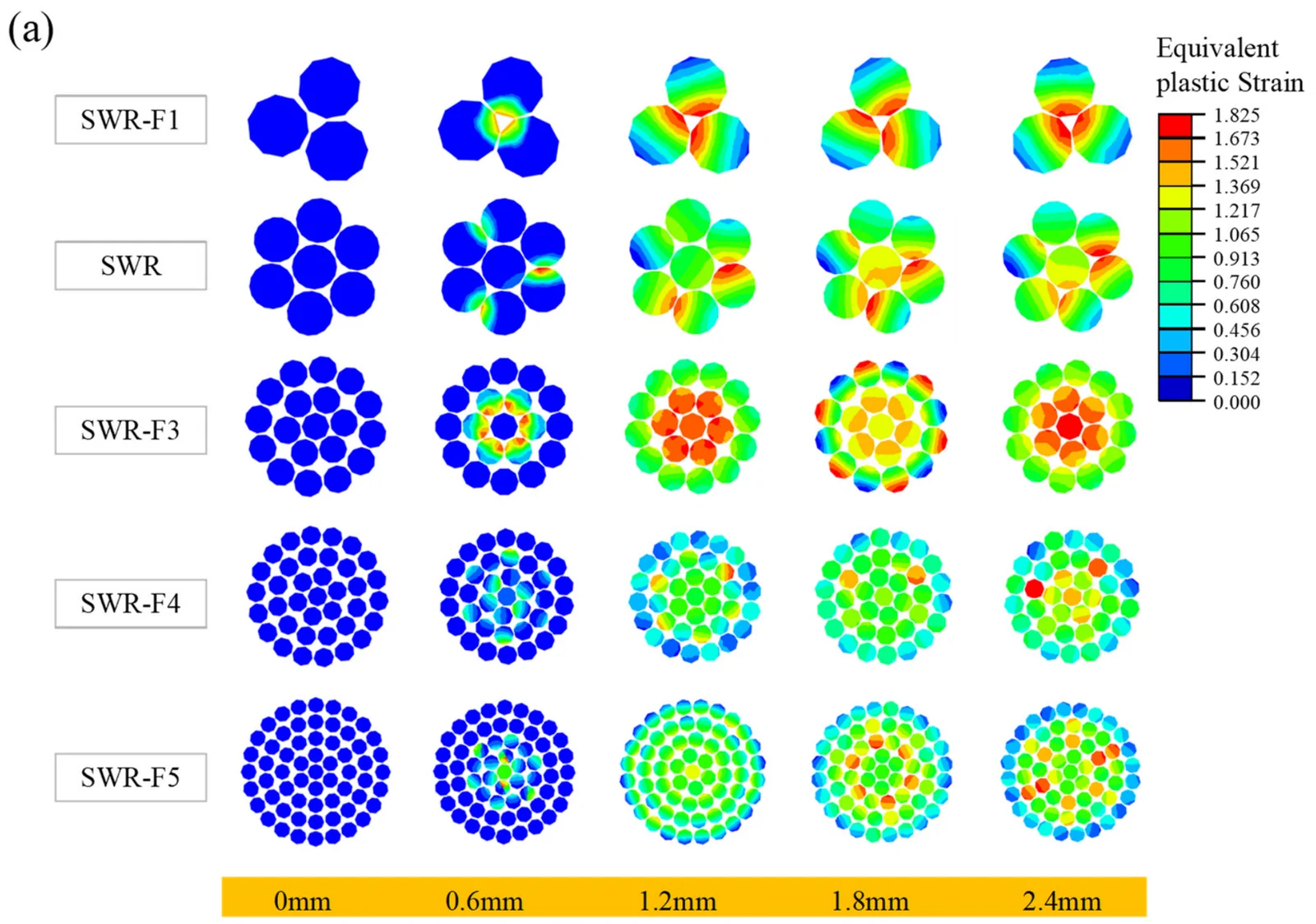

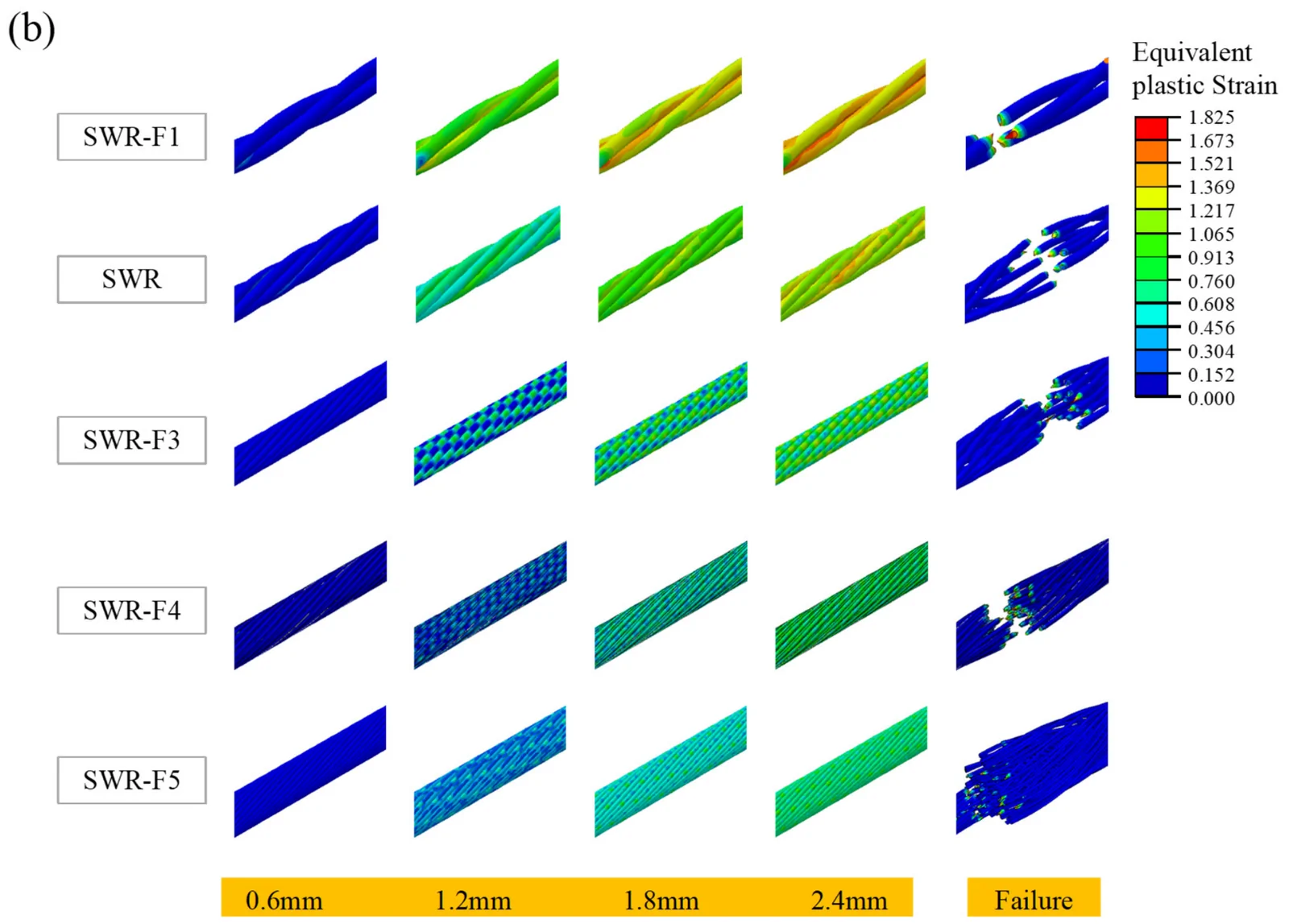
(**a**) Fracture cross-section strain distribution contours and (**b**) strain contour plots of SWR-F1, SWR, SWR-F3, SWR-F4 and SWR-F5. The color gradients represent local strain accumulation, highlighting the transition from uniform stress to localized yielding prior to fracture.

Figure 19a,b present the deformation patterns of SWR-F1, SWR, SWR-F3, SWR-F4, and SWR-F5, demonstrating how F governs strain evolution through coupled helical nesting constraints. Low-layer ropes exhibit gradual strain penetration due to sparse helical nesting, resulting in outer-dominated deformation. In contrast, high-layer ropes develop dense hierarchical architectures that improve load-sharing uniformity across multiple layers and enforce synchronized global strain evolution before fracture. This layer-dependent behavior reveals a hierarchical strategy for strength optimization and fracture control. Decreasing F promotes an uncoupled, outer-initiated fracture pattern due to sluggish strain permeation. Conversely, increasing F enforces a highly synchronized multi-layer load-sharing mechanism. This hierarchical architecture manages the risks of inner-layer microcracking and fundamentally alters the ultimate fracture mode, proving that layer count is a critical tuning variable for engineering custom tensile strength profiles and achieving an optimal force transmission efficiency up to 93.89%.

### 4.4. The Effect of Strand Structure S

**Figure 20:**
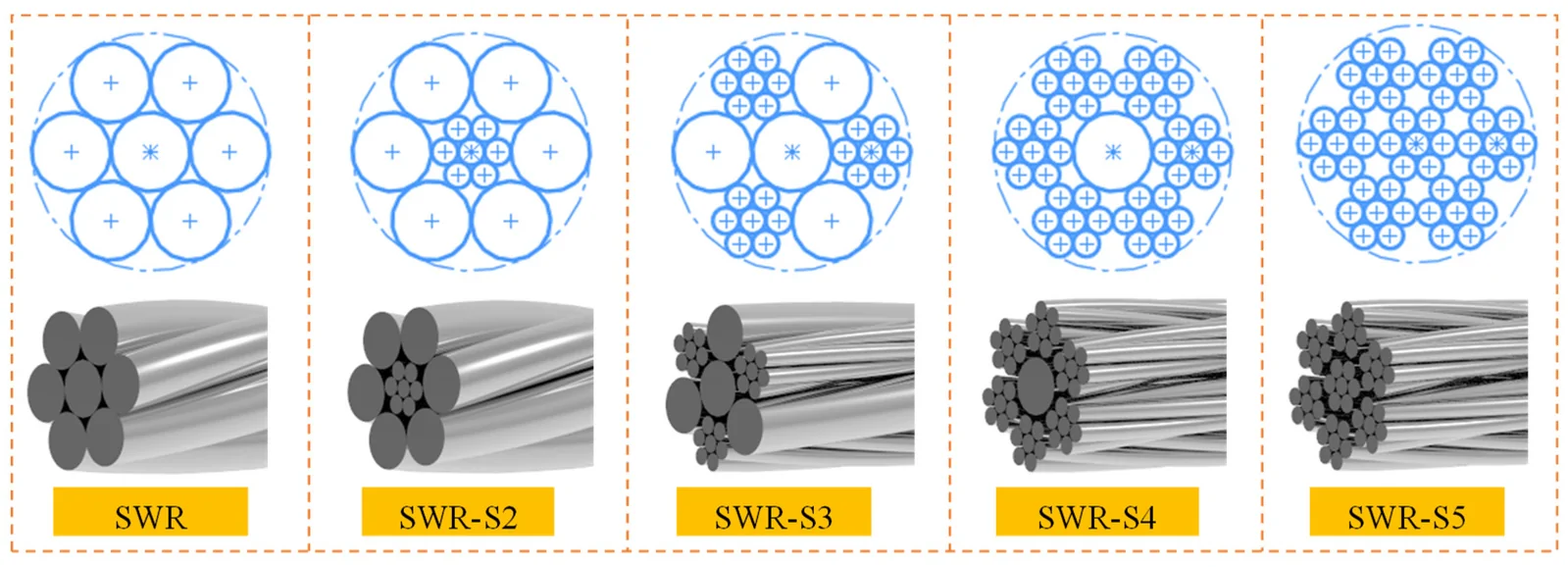
SWR consists of 7 wires in a two-layer twist: a monofilament core and a single-wire outer layer. SWR-S2 comprises 13 wires in two layers: a 1 × 7 core strand and a single-wire outer layer. SWR-S3 comprises 22 wires in two layers: a monofilament core and an outer layer with alternating single wires and 1 × 7 strands. SWR-S4 comprises 43 wires in two layers: a monofilament core and a 1×7 outer layer. SWR-S5 comprises 49 wires in two layers: a 1 × 7 core strand and a 1 × 7 outer layer.

This section systematically examines the effects of *S* on the tensile properties of SWRs while maintaining constant overall dimensions. Instead of treating the strand arrangement as a passive parameter, this configurational transition is utilized as a high-level structural tailoring method to adjust the macroscopic mechanical properties. The designed strand configurations are 1-1 × 7, (1 × 7)-1 × 7, 1-(1 × 7) × 3 + 1 × 3, 1-(1 × 7) × 6, and (1 × 7) × 6-(1 × 7) × 6, with the corresponding models named SWR, SWR-S2, SWR-S3, SWR-S4, and SWR-S5 respectively. The cross-sectional views of these models are shown in Figure 20, and their dimensional details are listed in Table 9.

**Table 9:** The detailed dimensions of these SWR-*S* models.

	*L* (mm)	*D* (mm)	Strand_core_	Strand_outer_	Twist Direction	SM (g)
SWR	50	1.44	Single-wire	Single-wire	Right	0.508
SWR-S2	50	1.44	1 × 7	Single-wire	Right Lang lay	0.474
SWR-S3	50	1.44	Single-wire	Single-wire and 1×7 alternated	Right Lang lay	0.444
SWR-S4	50	1.44	Single-wire	1 × 7	Right Lang lay	0.391
SWR-S5	50	1.44	1 × 7	1 × 7	Right Lang lay	0.374

**Figure 21:**
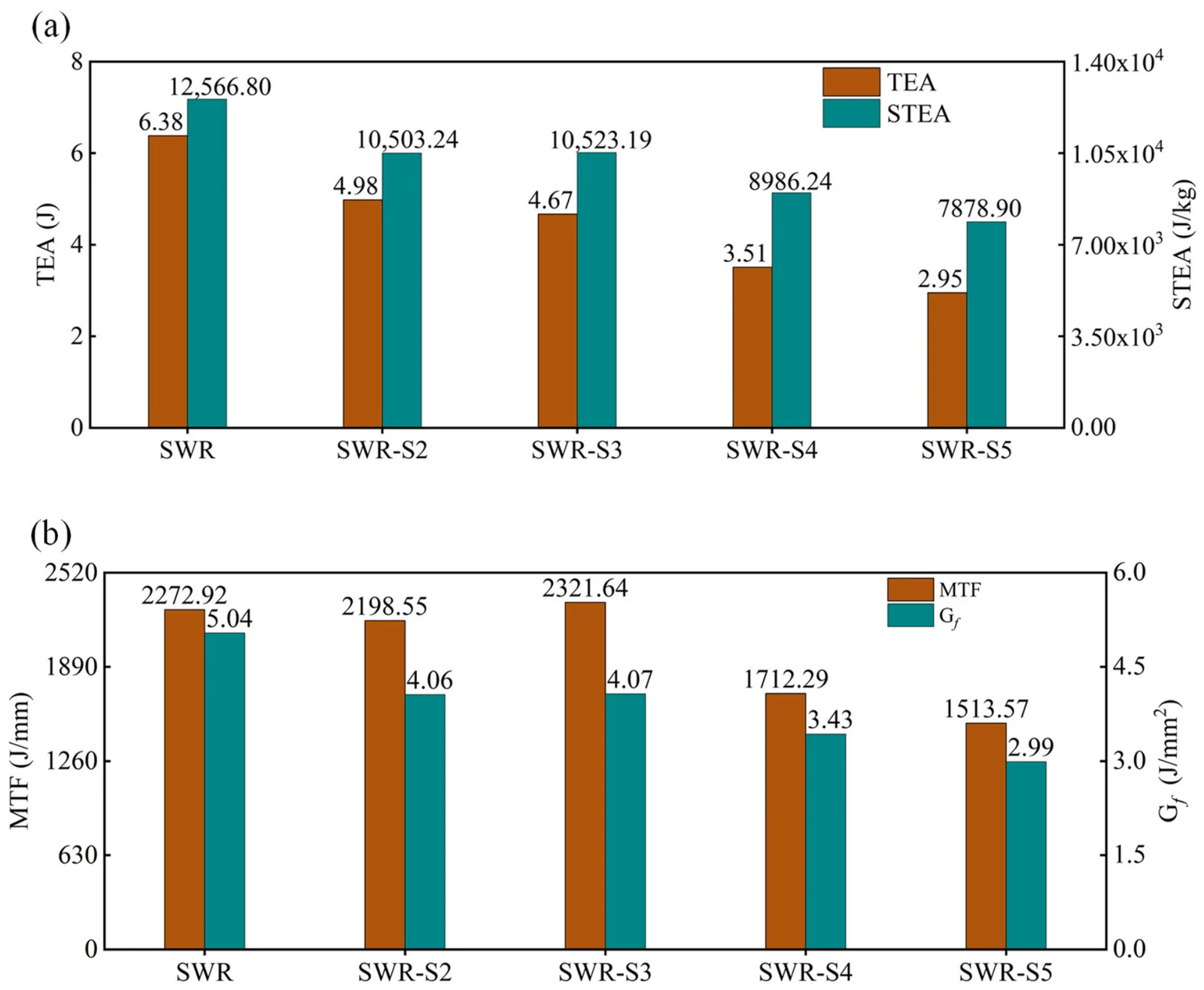
The histograms of (**a**) TEA and TSEA as well as (**b**) MTF and G*_f_* for SWR, SWR-S2, SWR-S3, SWR-S4 and SWR-S5.

Table 10 and Figure 21 present FEA results for SWR, SWR-S2, SWR-S3, SWR-S4, and SWR-S5. FEA was conducted on five distinct steel wire rope configurations. All configurations maintain a constant overall diameter *D* of 1.44 mm and a length L of 50 mm. The progressive transition from simple SWR to complex multi-strand designs SWR-S5 yields a 53.8% reduction in total energy absorption, decreasing from 6.38 J to 2.95 J. Consequently, monofilament structures demonstrate a 2.16-fold superiority in total tensile energy absorption under axial tensile loading. Specific energy absorption follows similar trends. It peaks at 12,566.80 J/kg for monofilament designs, which is 59.5% higher than that of SWR-S5. The hybrid SWR-S3 configuration achieves optimal multi-layer efficiency of 10,523.19 J/kg through balanced strand arrangement. Energy transfer efficiency displays non-monotonic behavior. It reaches 99.76% for SWR-S3’s hybrid architecture, an improvement of 16.2% over monofilament structures. It is critical to clarify that this high TFE indicates exceptional force-plateau uniformity and synchronized internal load sharing, rather than superior absolute strength or energy absorption. In fact, to achieve this near-perfect stability, SWR-S3 trades off absolute capacity, exhibiting a lower PTF (2327.33 N) and TEA (4.67 J) compared to the baseline SWR (PTF of 2648.58 N and TEA of 6.38 J). Then it declines to 81.51% in SWR-S5. This decline is due to intensified inter-strand friction. These results demonstrate that monofilament designs excel in applications requiring high specific energy absorption such as crane cables. Hybrid configurations optimize precision systems such as elevator ropes. Complex multi-strand structures are theoretically advantageous for weight-critical aerospace applications; however, transitioning these complex architectures to such rigorous fields will require future physical testing under dynamic impact, cyclic fatigue, and bending loads. These findings provide clear guidelines for performance-driven strand architecture selection.

The hybridization of strand configurations reveals highly localized optimization peaks in specific performance (Table 10). The SWR-S3 configuration stands out as exceptionally efficient; it not only achieves a near-perfect TFE of 99.76%, but it also records the highest specific strength (PTF/SM) among the hybrid models at 5241.73 N/g—marginally surpassing even the baseline SWR (5213.74 N/g). While its absolute TSEA (10,523.19 J/kg) is lower than the baseline, SWR-S3’s nominal ultimate stress remains highly competitive at 2030.74 MPa. Further complexity (e.g., SWR-S4 and SWR-S5) results in sharp declines across all normalized metrics, with the specific strength of SWR-S5 dropping to 4964.97 N/g. This pinpoint optimization proves that specific hybrid architectures (like SWR-S3) can successfully decouple load-sharing uniformity from the typical weight penalties associated with structural complexity.

**Figure 22:**
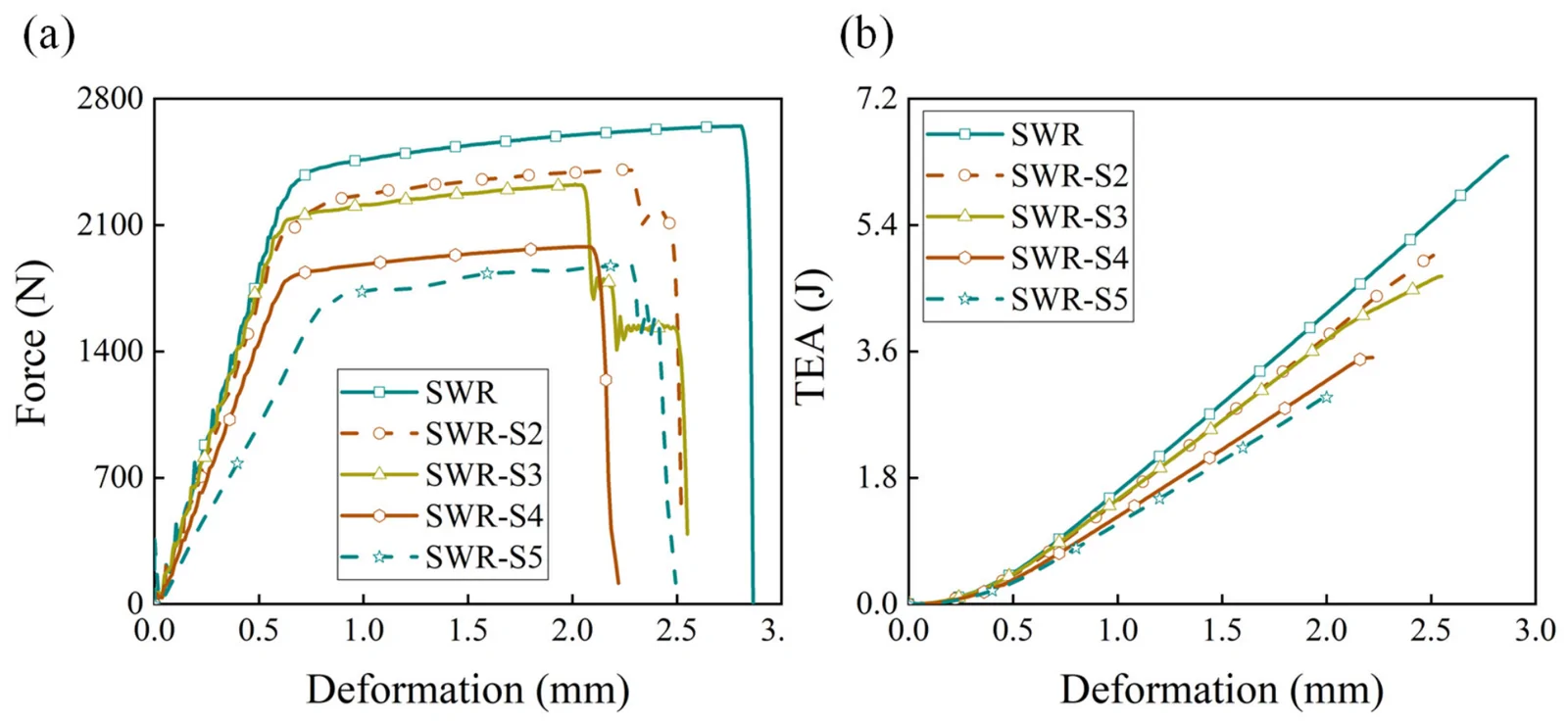
The results of (**a**) force– and (**b**) TEA–deformation curves of SWR, SWR-S2, SWR-S3, SWR-S4 and SWR-S5.

Tensile force–deformation and TEA–deformation curves for these SWR-S models are shown in Figure 22a,b. Forces for SWR, SWR-S2, SWR-S3, SWR-S4, and SWR-S5 increase approximately linearly with deformation, with SWR-S5 exhibiting a smaller initial slope and lower initial stiffness. In contrast, SWR shows a steeper force curve during loading, indicating superior stiffness. This contrast demonstrates that adjusting the sub-strand configuration is a highly effective means of mechanically tailoring the structural stiffness. For energy absorption, all models’ curves increase linearly, showing rapid energy absorption with deformation-evidence of good energy absorption capacity.

**Figure 23:**
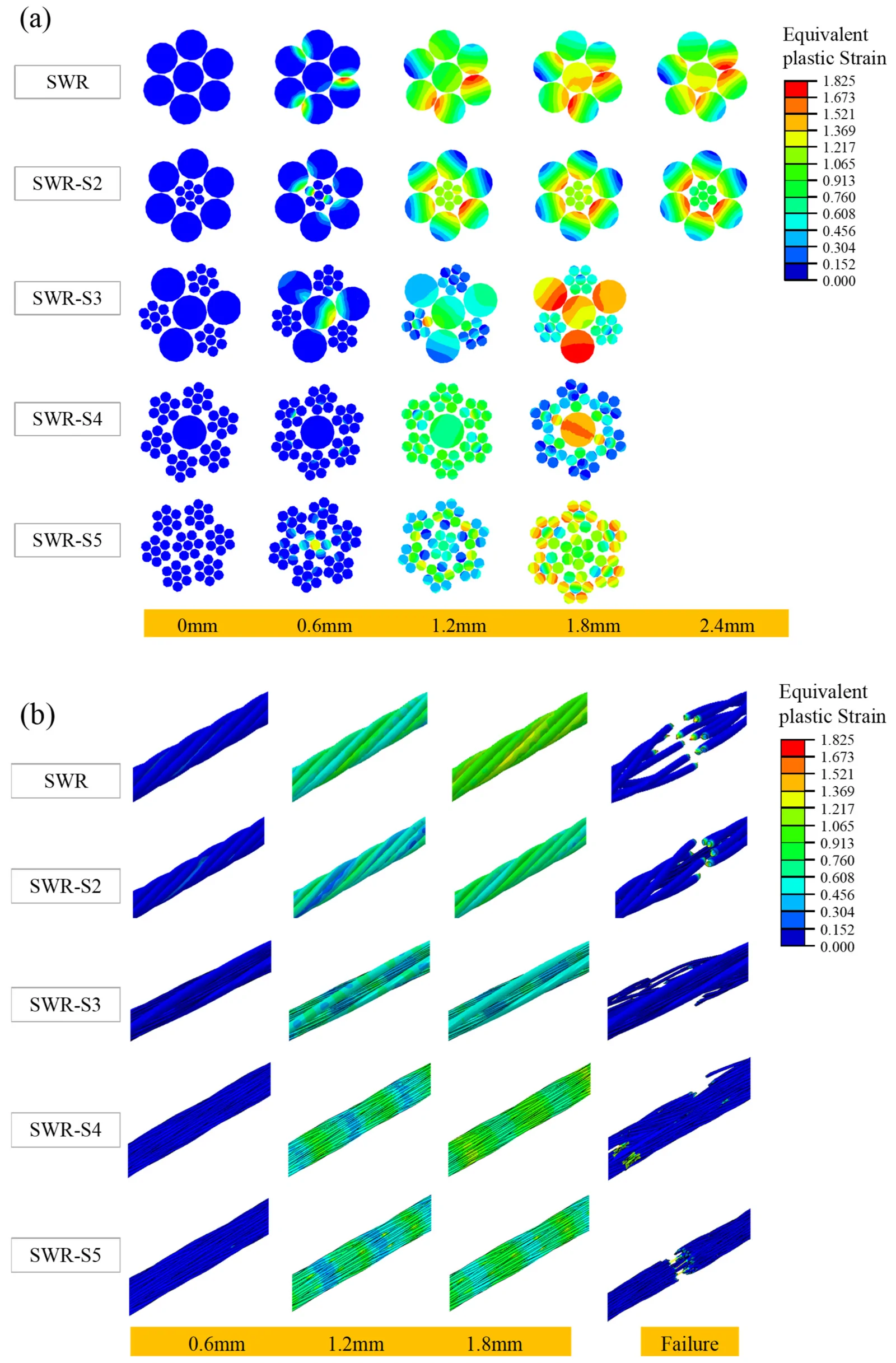
(**a**) Fracture cross-section strain distribution contours and (**b**) strain contour plots of SWR, SWR-S2, SWR-S3, SWR-S4 and SWR-S5. The color gradients represent local strain accumulation, highlighting the transition from uniform stress to localized yielding prior to fracture.

Figure 23a,b present the deformation patterns of SWR and its structural variants including SWR-S2, SWR-S3, SWR-S4 and SWR-S5. They demonstrate how S governs strain evolution through coupled wire exposure effects and substructural constraints. Simple designs exhibit gradual strain penetration, enabling outer-dominated yielding. In contrast, complex configurations develop dense substructural networks that trigger synchronous deformation patterns. This configuration-dependent behavior illustrates a complexity-induced transition in ultimate failure limits. Architectural complexity redistributes internal stresses, tuning the overall tensile strength and programming the fracture characteristic. Simpler designs favor progressive failure, whereas hybrid configurations like SWR-S3 enforce global strain synchronization prior to ultimate tensile failure, achieving a near-perfect force transmission of 99.76%. This proves that advanced strand architecture can be utilized to tailor the strength-to-stability ratio, ultimately facilitating the design of an ideal load plateau.

While the hybrid architectures (SWR-S2 to SWR-S5) demonstrate theoretically superior load-sharing and energy absorption, it is critical to address their practical manufacturability. The finite element models assume idealized, perfectly uniform geometries and pristine initial contact conditions. In reality, manufacturing a single concentric layer that alternates between solid single wires and 1 × 7 strands are highly complex. Because solid wires and multi-wire strands possess vastly different bending stiffnesses and tensioning behaviors, feeding them simultaneously through standard tubular stranding equipment would likely result in uneven tension, geometric distortion, or “bird-caging”. Consequently, SWR-S2 through SWR-S5 should currently be viewed as conceptual parametric designs that map the theoretical upper limits of hybrid load transfer. Physically realizing these architectures while maintaining the assumed contact constraints would require advanced planetary stranding machinery equipped with independent, highly calibrated tension-control systems for each distinct bobbin type.

### 4.5. Multi-Objective Comparative Analysis of SWR Configurations

#### 4.5.1. Multi-Objective Performance Evaluation

The mechanical performance of the investigated SWR configurations cannot be fully characterized by considering a single parameter, such as TFE. Although TFE provides important information regarding fracture completion and deformation coordination, a configuration with a high TFE value may not necessarily provide superior load-bearing capability or energy absorption performance. Therefore, a multi-objective evaluation method was introduced to assess the overall performance of different SWR architectures.

The evaluation considers five representative mechanical indicators, including PTF, TEA, TSEA, strength-to-weight ratio (PTF/SM), and TFE. These parameters characterize load-bearing capability, energy absorption capacity, lightweight efficiency, and fracture resistance. To eliminate the influence of different units and scales, all indicators were normalized using the min–max normalization method.

Subsequently, an equal-weight multi-objective performance index (MPI) was calculated:

(18) $$MPI=\frac{{PTF}^{*}+{TEA}^{*}+{TSEA}^{*}+{\left(PTF/SM\right)}^{*}+{TFE}^{*}}{5}$$

where the superscript * denotes the normalized value of each parameter. Equal weighting factors were assigned to all indicators.

The calculated MPI values and rankings are summarized in Table 11. The results demonstrate that the optimal configuration cannot be determined solely based on TFE. For example, SWR-S3 achieves the highest TFE value (99.76%), indicating excellent fracture efficiency. However, its relatively low PTF and TEA limit its overall mechanical performance. This result confirms that TFE alone is insufficient for ranking load-bearing structures. Therefore, although SWR-S3 exhibits superior fracture efficiency, it is not the optimal configuration for applications requiring high tensile capacity and energy absorption.

In contrast, SWR-D2.986 achieves the highest MPI value among all investigated configurations. Although SWR-D2.986 does not provide the highest strength-to-weight efficiency, its extremely high tensile capacity and energy absorption capability contribute significantly to its superior comprehensive performance. This configuration provides the highest peak tensile force (10,180.90 N) and tensile energy absorption (34.07 J), resulting from the increased cross-sectional area and enhanced load-sharing capability associated with the *D*. However, the increase in structural mass reduces its strength-to-weight efficiency compared with lightweight configurations.

SWR-F1 achieves the second-highest MPI value, followed by the baseline SWR and other optimized configurations with comparable MPI values. Although its absolute tensile force is lower than that of larger-diameter configurations, the reduced mass enables the highest specific strength among all investigated structures (5355.02 N/g), demonstrating its potential for lightweight applications where mass efficiency is prioritized rather than maximum load-bearing capability.

These results indicate that the structural optimization of SWRs involves inherent trade-offs between load-bearing capability, energy absorption, fracture efficiency, and weight efficiency. Increasing the core diameter effectively enhances the absolute tensile capacity and energy absorption, whereas lightweight configurations provide advantages in specific strength. Therefore, the selection of an optimal SWR architecture should be based on the targeted engineering requirements rather than a single performance indicator. The stiffness-to-mass ratio was not included as an optimization criterion in MPI because the present study focuses on balancing tensile capacity, energy absorption, and fracture efficiency. However, stiffness and specific stiffness were separately quantified to provide additional mechanical characterization.

#### 4.5.2. Structural Stiffness Comparison

**Figure 24:**
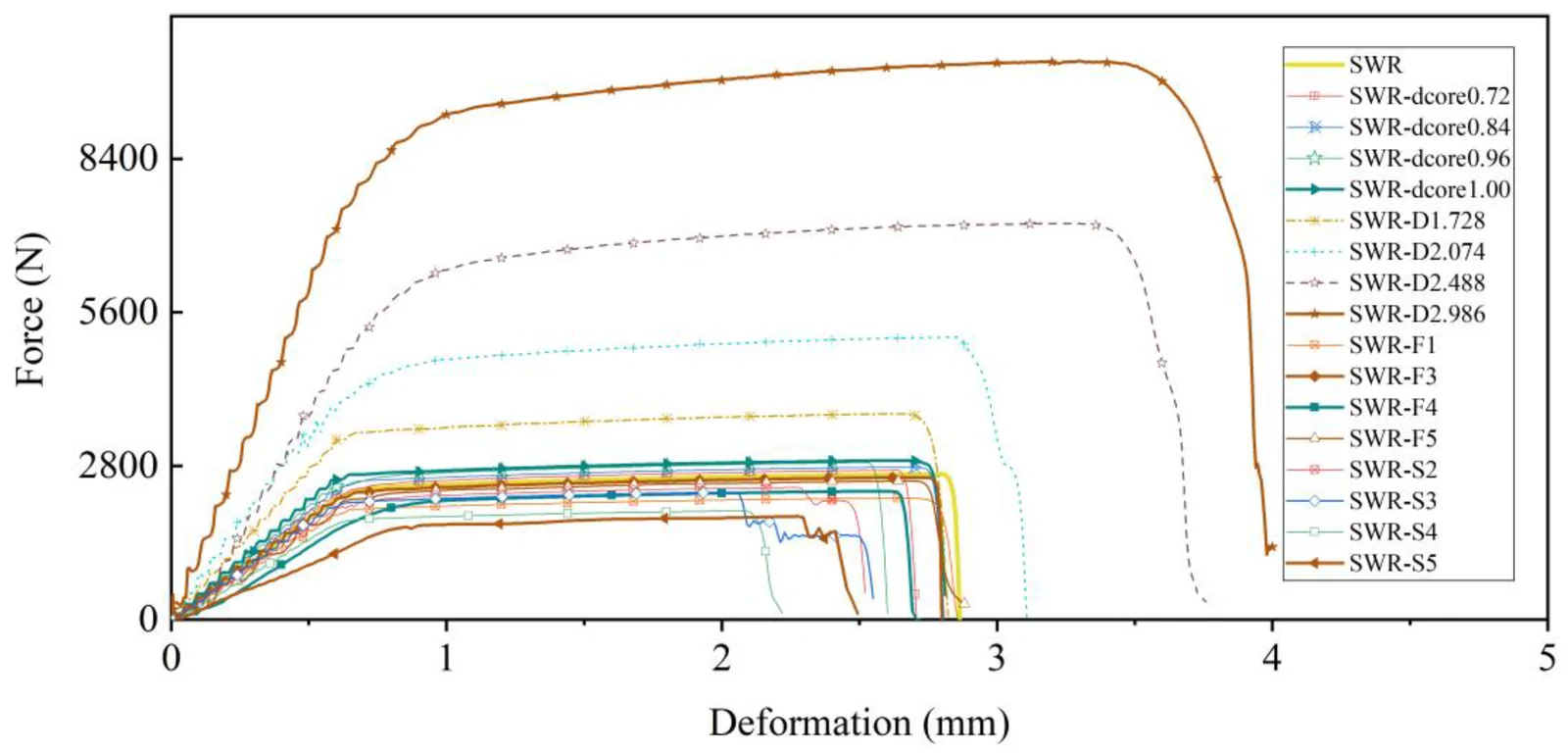
Force–deformation responses of representative SWR configurations.

As shown in Figure 24, the initial stiffness was calculated from the slope of the initial linear region of the force–deformation curves. The initial stiffness values and corresponding specific stiffness values are summarized in Table 12.

The results indicate that geometric modifications significantly affect both the absolute stiffness and stiffness-to-weight efficiency of SWRs. Among all investigated configurations, SWR-D2.986 exhibits the highest absolute initial stiffness of 12,525.93N/mm, which is attributed to the increased cross-sectional area and enhanced axial structural constraint associated with the enlarged rope diameter (*D* = 2.986 mm). However, evaluating normalized metrics reveals that SWR-D2.986 does not possess the highest specific stiffness. Its calculated specific stiffness is approximately 5422.48 N/mm·g^−1^, which is the lowest among the *D*-series models. Within the *D*-series, specific stiffness peaks at SWR-D1.728 (7406.57 N/mm·g^−1^) and decreases monotonically as the diameter expands. This mechanical trend occurs because the structural mass scales proportionally with the total cross-sectional area, whereas the initial linear tensile stiffness scales sub-linearly relative to mass due to internal helical compliance and inter-wire shear deformation across larger diameters. Consequently, while large-diameter ropes provide superior absolute rigidity, they experience diminishing returns in stiffness-to-mass efficiency.

Across all investigated structural architectures, the highest specific stiffness is achieved by SWR-S3 (8138.68 N/mm·g^−1^), followed by SWR-F1 (8064.11 N/mm·g^−1^). The superior specific stiffness of SWR-S3 originates from its hybrid multi-strand architecture, which successfully couples a low structural mass (SM = 0.444 g) with a highly synchronized, balanced axial load-transfer pathway (K = 3613.57 N/mm).

Increasing *d*_core_ systematically improves both absolute initial stiffness and specific stiffness. For example, the absolute stiffness of SWR-dcore1.00 increases to 4323.09 N/mm compared with 3707.63 N/mm for the baseline SWR, while its specific stiffness reaches 7624.50 N/mm·g^−1^ (compared with 7298.48 N/mm·g^−1^ for the baseline). This demonstrates that enlarging the central core reinforces internal radial support and enhances axial load-transfer efficiency without causing severe mass penalties.

In contrast, the influence of F is non-monotonic. SWR-F3 exhibits higher stiffness than SWR-F4, indicating that a moderate number of wire layers balances internal support against structural complexity. The four-layer architecture (SWR-F4) introduces pronounced helical compliance and inter-wire sliding, which significantly dampens the initial elastic stiffness response (K = 3095.45 N/mm, K/SM = 6266.09 N/mm·g^−1^). Although the five-layer configuration (SWR-F5) recovers some stiffness (K = 3350.53 N/mm, K/SM = 6796.21 N/mm·g^−1^) due to denser internal structural constraints, its stiffness remains lower than that of the optimal three-layer structure. Similarly, within the S-series configurations, increasing sub-strand complexity generally reduces initial stiffness, with SWR-S5 exhibiting the lowest stiffness among the S-series models (2096.17 N/mm) attributable to more complex internal deformation modes and inter-strand contact compliance.

## 5. Conclusions

This study establishes and experimentally validates an FEA framework for baseline single-wire and 1 × 7 SWR models via axial tensile tests. Extending beyond conventional performance evaluation methods, we systematically investigated how to adjust the tensile strength and fracture properties of SWRs by varying parameters. The key tuning parameters analyzed include *d*_core_, *D*, *F*, and *S*. The primary numerical findings are summarized as follows:

(1) The results demonstrate that increasing *d*_core_ and the outer wire count significantly enhances the ultimate tensile strength and delays structural fracture, with the SWR-d_core_1.00 variant achieving the highest PTF and load-bearing capacity within the core-diameter parametric series. Across all investigated configurations, the large-diameter SWR-D2.986 achieves the highest absolute PTF (10,180.90 N), a result that is fundamentally associated with structural geometric scaling.
(2) Regarding specific energy absorption, the geometric trends diverge: within the core-diameter series, the baseline configuration with a smaller $d_{core}$ maintains superior specific energy efficiency; however, in the *D*-series, the specific energy absorption exhibits a non-monotonic trend, initially decreasing before reaching a maximum at the largest diameter (SWR-D2.986). Furthermore, multi-layer fine-wire-interlaced structures such as SWR-F5 and hybrid strand configurations such as SWR-S3 demonstrate outstanding force transmission stability.
(3) Structural parameter optimization enables SWRs to achieve an optimal tunable range, balancing tensile strength limits, energy absorption, and lightweight performance. Specifically, large-diameter and robust-core models including SWR-d_core_1.00 and SWR-D2.986 are engineered for extreme heavy-load conditions. Low-layer and small-diameter models including SWR-F1 and SWR are applicable to lightweight designs, whereas complex hierarchical configurations including SWR-F5 and SWR-S3 are ideal for high-stability scenarios requiring controlled failure.

Ultimately, this study demonstrates the potential of complementing traditional material enhancement strategies with active geometric parametrization. It provides a conceptual theoretical baseline for exploring application-specific cables with custom-tailored tensile strength profiles and predictable failure behaviors.

Despite these promising insights, several numerical limitations remain: (1) idealized geometries, which neglect manufacturing-induced residual stresses, initial distortions, and tension imbalances; (2) limited experimental validation, as tests were restricted to quasi-static tension on single wires and 1 × 7 strands, leaving complex SWR-F and SWR-S configurations numerically extrapolated; (3) simplified contact mechanics, using a constant static friction coefficient rather than dynamic, wear-dependent helical friction; and (4) mesh sensitivity, where absolute fracture energy predictions remain sensitive to element density despite uniform meshing. To bridge these gaps and advance toward industrial implementation, future research will focus on four comprehensive areas: targeted prototype testing by fabricating and experimentally evaluating key architectures—specifically SWR-S3 for its 99.76% TFE stability and SWR-$d_{\mathrm{core}}1.00$ for its enhanced peak tensile capacity; friction sensitivity analysis by combining parametric simulations and tribological tests to quantify how surface roughness, wear, and grease degradation affect strain synchronization and load-bearing limits; service-relevant loading by extending models beyond quasi-static tension to incorporate cyclic bending fatigue, tension–torsion coupling, and dynamic impact loading; and fracture-surface characterization by using SEM and microstructural analyses to correlate physical void nucleation and micro-crack coalescence with numerical damage evolution criteria.

## Figures and Tables

**Table 1:** The material parameters of steel wire rope.

	**Ductile Damage**			
Density	Fracture strain	Stress Triaxiality	Strain Rate	Damage Evolution (mm)
7.85 × 10^3^	0.85	0.333	0	0.1
**Elastic**		**Plastic (Simplified Power Law)**		
Young’s modulus (GPa)	Poisson’s ratio	A (MPa)	B (MPa)	n
174.968	0.3	2012.85	2774.65	0.65667

**Table 2:** Numerical values of single wire and 1 × 7 steel wire rope tensile experiment and FEA.

		SM (g)	TEA (J)	TSEA (J/kg)	MTF (N)	*G*_f_ (J/mm^2^)	PTF (N)	TFE
Single wire	FEA	0.071	0.82887	11,674.23	334.76	4.58	391.88	85.42%
	Experiment	0.071	0.83270	11,728.17	309.67	4.60	401.00	77.22%
	Error	0	−0.46%	−0.46%	8.10%	−0.46%	−2.27%	10.62%
1 × 7 steel wire rope	FEA	0.508	6.38393	12,566.80	2272.93	5.04	2648.58	85.82%
	Experiment	0.508	6.52281	12,840.18	2363.34	5.15	2651.00	89.15%
	Error	0	−2.13%	−2.13%	−3.83%	−2.13%	−0.09%	−3.74%

Note: “Error” is the difference between simulation and experimental results, and its expression is “Error = (FEA − experiment)/experiment”.

**Table 4:** The simulation results of SWR-*d*_core_ models.

	SM (g)	A_0_ (mm^2^)	TEA (J)	TSEA (J/kg)	G_f_ (J/mm^2^)	MTF (N)	PTF (N)	PTF/SM (N/g)	PTF/A0 (MPa)	TFE
SWR	0.508	1.27	6.38	12,566.80	5.04	2272.92	2648.58	5213.74	2090.95	85.82%
SWR-d_core_0.72	0.530	1.32	6.17	11,634.24	4.66	2341.48	2724.75	5141.04	2059.15	85.93%
SWR-d_core_ 0.84	0.539	1.35	6.56	12,168.66	4.87	2447.17	2779.37	5156.53	2063.68	88.05%
SWR-d_core_ 0.96	0.560	1.40	6.19	11,061.28	4.42	2447.49	2863.91	5114.13	2042.14	85.46%
SWR-d_core_1.00	0.567	1.42	6.94	12,231.47	4.88	2615.10	2903.33	5120.51	2043.48	90.07%

**Table 6:** The simulation results of SWR-*D* models.

	SM (g)	A_0_ (mm^2^)	TEA (J)	TSEA (J/kg)	G*_f_* (J/mm^2^)	MTF (N)	PTF (N)	PTF/SM (N/g)	PTF/A_0_ (MPa)	TFE
SWR	0.508	1.27	6.38	12,566.80	5.04	2272.92	2648.58	5213.74	2090.95	85.82%
SWR-D1.728	0.737	1.82	8.91	12,091.29	4.89	2407.82	3752.63	5091.76	2057.32	64.16%
SWR-D2.074	1.070	2.63	13.18	12,321.77	5.02	3128.74	5147.82	4811.05	1959.87	60.78%
SWR-D2.488	1.570	3.78	21.88	13,938.35	5.76	4080.81	7221.80	4599.87	1909.36	56.51%
SWR-D2.986	2.310	5.06	34.07	14,747.16	6.73	6631.69	10,180.90	4407.32	2012.17	65.14%

**Table 8:** The simulation results of SWR-*F* models.

	SM (g)	A_0_ (mm^2^)	TEA (J)	TSEA (J/kg)	G*_f_* (J/mm^2^)	MTF (N)	PTF (N)	PTF/SM (N/g)	PTF/A_0_ (MPa)	TFE
SWR-F1	0.414	1.04	5.33	12,871.77	5.13	1989.37	2216.98	5355.02	2134.10	89.73%
SWR	0.508	1.27	6.38	12,566.80	5.04	2272.92	2648.58	5213.74	2090.95	85.82%
SWR-F3	0.498	1.24	6.13	12,303.77	4.95	2290.54	2589.25	5199.30	2091.92	88.64%
SWR-F4	0.494	1.23	5.05	10,229.26	4.11	1964.21	2343.15	4743.22	1907.49	83.83%
SWR-F5	0.493	1.23	5.93	12,038.15	4.84	2194.82	2337.75	4741.89	1906.07	93.89%

**Table 10:** The simulation results of SWR-*S* models.

	SM (g)	A_0_ (mm^2^)	TEA (J)	TSEA (J/kg)	G*_f_* (J/mm^2^)	MTF (N)	PTF (N)	PTF/SM (N/g)	PTF/A_0_ (MPa)	TFE
SWR	0.508	1.27	6.38	12,566.80	5.04	2272.92	2648.58	5213.74	2090.95	85.82%
SWR-S2	0.474	1.23	4.98	10,503.24	4.06	2198.55	2407.93	5080.02	1963.29	91.30%
SWR-S3	0.444	1.15	4.67	10,523.19	4.07	2321.64	2327.33	5241.73	2030.74	99.76%
SWR-S4	0.391	1.03	3.51	8986.24	3.43	1712.29	1980.31	5064.73	1931.23	86.47%
SWR-S5	0.374	0.99	2.95	7878.90	2.99	1513.57	1856.90	4964.97	1884.79	81.51%

**Table 11:** Multi-objective performance evaluation and ranking of different steel wire rope configurations based on MPI.

Configuration	PTF*	TEA*	TSEA*	PTF/SM*	TFE*	MPI	Rank
SWR-D2.986	1.000	1.000	1.000	0.000	0.200	0.640	1
SWR-F1	0.043	0.076	0.727	1.000	0.768	0.523	2
SWR	0.095	0.110	0.683	0.851	0.678	0.483	3
SWR-d_core_1.00	0.126	0.128	0.634	0.753	0.776	0.483	4
SWR-F3	0.088	0.102	0.644	0.836	0.743	0.483	5
SWR-S3	0.057	0.055	0.385	0.880	1.000	0.475	6
SWR-d_core_ 0.84	0.111	0.116	0.625	0.791	0.729	0.474	7
SWR-D2.488	0.645	0.608	0.882	0.203	0.000	0.468	8
SWR-d_core_ 0.72	0.104	0.103	0.547	0.774	0.680	0.442	9
SWR-d_core_ 0.96	0.121	0.104	0.463	0.746	0.669	0.421	10
SWR-S2	0.066	0.065	0.382	0.710	0.804	0.406	11
SWR-F5	0.058	0.096	0.606	0.353	0.864	0.395	12
SWR-D1.728	0.228	0.192	0.613	0.722	0.177	0.386	13
SWR-D2.074	0.395	0.329	0.647	0.426	0.099	0.379	14
SWR-S4	0.015	0.018	0.161	0.694	0.693	0.316	15
SWR-F4	0.058	0.067	0.342	0.354	0.632	0.291	16
SWR-S5	0.000	0.000	0.000	0.588	0.578	0.233	17

Note: All parameters were normalized using the min–max normalization method. MPI was calculated using equal weights for all selected indicators.

**Table 12:** Initial stiffness and specific stiffness of different SWR configurations.

Configuration	Initial Stiffness K (N/mm)	Specific Stiffness K/SM (N/mm·g^−1^)	Configuration	Initial Stiffness K (N/mm)	Specific Stiffness K/SM (N/mm·g^−1^)
SWR	3707.63	7298.49	SWR-F1	3338.54	8064.11
SWR-d_core_0.72	3795.14	7160.63	SWR-F3	3766.18	7562.60
SWR-d_core_0.84	4097.22	7601.53	SWR-F4	3095.45	6266.09
SWR-d_core_0.96	4125.02	7366.11	SWR-F5	3350.53	6796.21
SWR-d_core_1.00	4323.09	7624.50	SWR-S2	3445.80	7269.63
SWR-D1.728	5458.64	7406.57	SWR-S3	3613.57	8138.68
SWR-D2.074	7069.77	6607.26	SWR-S4	3065.47	7840.07
SWR-D2.488	8995.82	5729.82	SWR-S5	2096.17	5604.72
SWR-D2.986	12,525.93	5422.48	-	-	-

## Data Availability

The original contributions presented in this study are included in the article. Further inquiries can be directed to the corresponding author.

## Abbreviations

The following abbreviations are used in this manuscript:

SWRs	steel wire ropes
TFE	tensile force efficiency
TEA	tensile energy absorption
TSEA	tensile specific energy absorption
*G*_f_	fracture energy
PTF	peak tensile force
MTF	mean tensile force
SM	structural mass
*d*_core_	core diameter
*D*	overall rope diameter
*F*	number of layers
*S*	strand configuration
SWR	SWR model with *d*_core_ of 0.48 mm
SWR-d_core_0.72	SWR model with *d*_core_ of 0.72mm
SWR-d_core_0.84	SWR model with *d*_core_ of 0.84mm
SWR-d_core_0.96	SWR model with *d*_core_ of 0.96mm
SWR-d_core_1.00	SWR model with *d*_core_ of 1.00mm
SWR-D1.728	SWR model with *D* of 1.728mm
SWR-D2.074	SWR model with *D* of 2.074mm
SWR-D2.488	SWR model with *D* of 2.488mm
SWR-D2.986	SWR model with *D* of 2.986mm
SWR-F1	SWR model with *F* of 1
SWR-F3	SWR model with *F* of 3
SWR-F4	SWR model with *F* of 4
SWR-F5	SWR model with *F* of 5
SWR-S2	SWR model comprises 13 wires in two layers: a 1 × 7 core strand and a single-wire outer layer.
SWR-S3	SWR model comprises 22 wires in two layers: a monofilament core and an outer layer with alternating single wires and 1 × 7 strands.
SWR-S4	SWR model comprises 43 wires in two layers: a monofilament core and a 1 × 7 outer layer.
SWR-S5	SWR model comprises 49 wires in two layers: a 1 × 7 core strand and a 1 × 7 outer layer.
